# COVID-19 Pandemic Waves and Mortality Among Patients on Kidney Replacement Therapy

**DOI:** 10.1016/j.ekir.2022.06.007

**Published:** 2022-06-20

**Authors:** Priya Vart, Kitty J. Jager, Miha Arnol, Raphaël Duivenvoorden, Casper F.M. Franssen, Marc Groeneveld, Marc H. Hemmelder, Fanny Lepeytre, Thomas Malfait, Karsten Midtvedt, Sandip Mitra, Carme Facundo, Marlies Noordzij, Carlos C. Reina, Seda Safak, Nestor Toapanta, Luuk B. Hilbrands, Ron T. Gansevoort

**Affiliations:** 1 Department of Internal Medicine, University Medical Center Groningen, University of Groningen, Groningen, The Netherlands; 2 Department of Clinical Pharmacy & Pharmacology, University Medical Center Groningen, Groningen, The Netherlands; 3 ERA Registry, Department of Medical Informatics, Amsterdam University Medical Center, Amsterdam Public Health Research Institute, Amsterdam, The Netherlands; 4 Department of Nephrology, University Medical Center Ljubljana; Medical Faculty, University of Ljubljana, Ljubljana, Slovenia; 5 Department of Nephrology, Radboud University Medical Center, Nijmegen, The Netherlands; 6 Haaglanden Medical Center, the Hague, The Netherlands; 7 Division on Nephrology, Department of Internal Medicine, Maastricht University Medical Center; CARIM School for Cardiovascular Research, University Maastricht, Maastricht, The Netherlands; 8 Claude Galien Hospital Ramsay Santé, Quincy-sous-Sénart, France; 9 Department of Nephrology, AZ Delta, Roeselare, Belgium; 10 Department of Transplantation Medicine, Oslo University Hospital – Rikshospitalet, Olso, Norway; 11 Department of Renal Medicine, Manchester University Hospitals, Manchester Academy of Health Sciences Center, University of Manchester, Manchester, UK; 12 Fundació Puigvert, Barcelona, Spain; 13 Toledo University Hospital, Toledo, Spain; 14 Division of Nephrology, Department of Internal Medicine, Istanbul Faculty of Medicine, Istanbul University, Istanbul, Turkey; 15 Vall d’Hebron University Hospital, Barcelona, Spain

**Keywords:** COVID-19, dialysis, kidney, mortality, pandemic wave, transplant

## Introduction

In the first year of the COVID-19 pandemic, many countries observed a 2-wave pattern in the daily reported cases, namely a first wave between March 2020 and July 2020, and thereafter a second wave between August 2020 and February 2021. In Europe, the first wave corresponded with the spring and summer seasons, and the second wave with the autumn and winter seasons. General population data from several countries suggested a lower risk of mortality in the second wave compared with the first wave.^1^, ^2^, ^3^ Some of the potential explanations for this include the increased identification of young individuals with COVID-19,^1^ improved test capacity leading to the identification of less severe cases,^2^ and improved patient management^3^ during the second wave compared with the first wave.

A number of studies compared mortality in the first and second waves among patients receiving kidney replacement therapy. These studies were hampered by the fact that they were single center by design and consequently had a small sample size.^4^ Furthermore, they lacked information on key patient and disease-related characteristics including comorbidities, the reason for COVID-19 screening, and disease symptoms.^4^, ^5^, ^6^, ^7^, ^8^, ^9^

Using data from the largest European database of kidney replacement therapy patients with COVID-19, that was collected at multiple centers across Europe and has detailed information on key covariates, we compared mortality between the first and second pandemic waves among dialysis patients and kidney transplant recipients with COVID-19. Secondly, we examined potential reasons for any observed differences in mortality between the 2 waves.

## Results

### Dialysis Patients

Of a total of 3004 dialysis patients with COVID-19, 1253 (41.7%) were recorded in the first wave and 1751 (58.3%) in the second wave (Supplementary Table S1 and Supplementary Figure S1). Patients in the second wave were older with no difference in distribution of males and females between the 2 waves.

**Figure 1:**
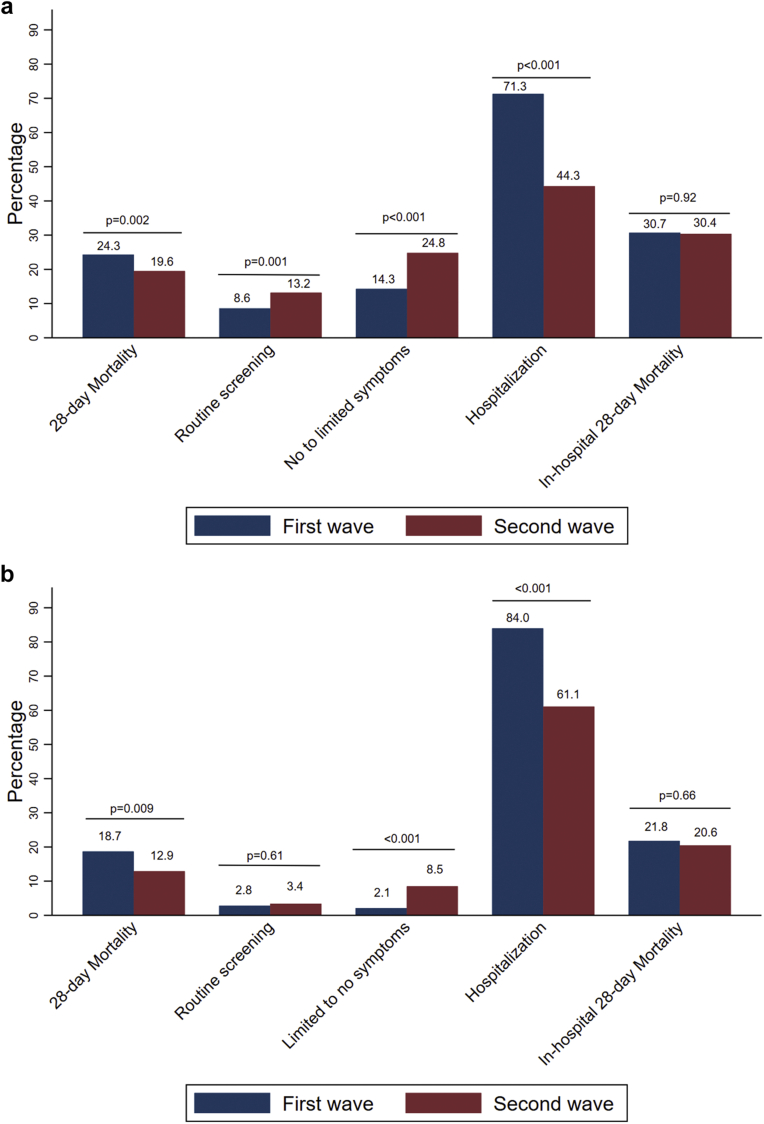
Key characteristics and outcomes by pandemic waves (first and second) in dialysis patients (panel a) and kidney transplant recipients (panel b). Dialysis patients (a). Kidney transplant recipient (b).

In the second wave, when compared with the first wave, the crude 28-day mortality rate was lower (19.6% vs. 24.3%, *P* = 0.002) (Figure 1a) and cumulative survival higher (*P* < 0.001) (Supplementary Figure S2). In the second wave, patients were more often identified through routine screening for COVID-19 and consequently the proportion of patients with limited or no symptoms at time of detection was higher compared with patients in the first wave (Figure 1a). Hospitalization rate was significantly lower in the second wave, whereas in-hospital mortality was similar in the 2 waves (Figure 1a), as was in-hospital cumulative survival at day 28 (*P* = 0.52) (Supplementary Figure S2).

In Cox models, the second wave (vs. the first wave) was associated with a lower risk of mortality in a crude (hazard ratio = 0.77, 95% CI: 0.66–0.89, *P* = 0.001) but not in a fully adjusted model (hazard ratio = 0.93, 95% CI: 0.79–1.10, *P* = 0.38) (Table 1). When hospitalized and nonhospitalized patients were analyzed separately, it showed that in both subpopulations the second wave (vs. the first wave) was not associated with a lower risk of mortality in the crude model or in the fully adjusted model (Table 1).

**Table 1:** Twenty-eight day mortality in the second pandemic wave (vs. the first wave) in total population and by hospitalization status among dialysis patients and kidney transplant recipients (presented are hazard ratios with 95% CIs)

Models	Dialysis patients (*N* = 3004)			Kidney transplant recipients (*N* = 1035)		
	First wave (*n* = 1253)	Second wave (*n* = 1751)	*P*-value	First wave (*n* = 475)	Second wave (*n* = 560)	*P*-value
Mortality, *n* (%)	304 (24.3)	344 (19.6)	0.002	89 (18.7)	72 (12.9)	0.009
Model 1	Ref.	0.77 (0.66–0.89)	0.001	Ref.	0.66 (0.48–0.90)	0.008
Model 2	Ref.	0.69 (0.59–0.80)	<0.001	Ref.	0.79 (0.58–1.08)	0.13
Model 3	Ref.	0.74 (0.63–0.86)	<0.001	Ref.	0.85 (0.62–1.16)	0.30
Model 4	Ref.	0.77 (0.65–0.90)	0.001	Ref.	0.82 (0.59–1.12)	0.21
Model 5	Ref.	0.78 (0.66–0.92)	0.003	Ref.	0.82 (0.58–1.15)	0.25
Model 6	Ref.	0.93 (0.79–1.10)	0.38	Ref.	0.95 (0.68–1.33)	0.76

	Hospitalized (*n* = 741)					
	First wave (*n* = 893)	Second wave (*n* = 775)	*P*-value	First wave (*n* = 399)	Second wave (*n* = 342)	*P*-value
Mortality, *n* (%)	274 (30.7)	236 (30.4)	0.92	87 (21.8)	70 (20.5)	0.66
Model 1	Ref.	0.94 (0.79–1.12)	0.52	Ref.	0.92 (0.67–1.26)	0.59
Model 2	Ref.	0.86 (0.72–1.03)	0.10	Ref.	0.97 (0.71–1.34)	0.87
Model 3	Ref.	0.89 (0.74–1.05)	0.17	Ref.	1.01 (0.73–1.38)	0.97
Model 4	Ref.	0.89 (0.74–1.06)	0.19	Ref.	0.99 (0.71–1.36)	0.93
Model 5	Ref.	0.88 (0.74–1.05)	0.18	Ref.	0.97 (0.69–1.36)	0.86

	Nonhospitalized (*n* = 294)					
	First wave (*n* = 360)	Second wave (*n* = 976)	*P*-value	First wave (*n* = 76)	Second wave (*n* = 218)	*P*-value
Mortality, *n* (%)	30 (8.3)	108 (11.1)	0.14	2 (2.6)	2 (0.9)	0.27
Model 1	Ref.	1.34 (0.89–2.00)	0.16	Ref.	NR	
Model 2	Ref.	1.11 (0.74–1.66)	0.62	Ref.	NR	
Model 3	Ref.	1.16 (0.77–1.75)	0.48	Ref.	NR	
Model 4	Ref.	1.11 (0.72–1.71)	0.63	Ref.	NR	
Model 5	Ref.	1.10 (0.70–1.73)	0.68	Ref.	NR	

NR, not reliable (due to too few events); Ref., reference.

Model 1: crude. Model 2: model 1 + age, and sex. Model 3: model 2 + reason for screening, and presence of no to limited symptoms. Model 4: model 3 + smoking, hypertension, diabetes mellitus, chronic lung disease, heart failure, chronic artery disease, autoimmune disease, malignancy, and frailty score. Model 5: model 4 + cough, shortness of breath, fever, sore throat, oxygen saturation, pulse, temperature, systolic blood pressure, diastolic blood pressure, lymphocytes, and C-reactive protein. Model 6: model 5 + hospitalization.

### Kidney Transplant Recipients

Among the 1035 kidney transplant recipients with COVID-19, 475 (45.9%) were recorded in the first wave and 560 (54.1%) in the second wave (Supplementary Table S1 and Supplementary Figure S1). Kidney transplant recipients were younger in the second wave compared with the first wave.

Similar to dialysis patients, the total 28-day mortality was lower in the second wave (12.9% vs. 18.7%, *P* = 0.009) (Figure 1b), and cumulative survival was higher in the second wave (*P* = 0.007) (Supplementary Figure S2). Percentages of patients identified through routine screening were similar though the proportion of patients with limited or no symptoms detected during the second wave was higher compared with patients in the first wave (Figure 1b). The hospitalization rate was lower in the second wave, whereas in-hospital mortality was similar in the 2 waves (Supplementary Figure S2).

The second wave (vs. the first wave) was associated with a lower risk of mortality only in the crude model. After adjusting for age and sex, this association was not statistically significant, whereas in the fully adjusted model, the hazard ratio for the risk of mortality was even close to unity (Table 1). Among hospitalized patients, pandemic wave was not associated with mortality, and among nonhospitalized patients the number of deaths was too small to reliably investigate this association (Table 1).

## Discussion

Because the testing capacity increased over time during the pandemic, screening for SARS-CoV-2 was more intense during the second wave than in the first wave.S1 Accordingly, in our study the proportion of patients with limited or no symptoms was higher and rates of crude mortality were lower in the second wave compared with the first wave. Importantly, when mortality was investigated among patients with comparable disease severity, (i.e., by hospitalized status) mortality in both waves was similar in dialysis patients as well as in kidney transplant recipients. These findings were consistent after accounting for a possible between-country difference in patient and disease characteristics, and patient management, and when using different cut-off dates for the distinction between the first and the second wave (Supplementary Tables S1–S16, Supplementary Figures S3–S6 and Supplementary Results).

Because of more intense screening for SARS-CoV2 during the second wave, there was an increased likelihood of identifying patients with limited to no symptoms with a number of them being diagnosed earlier in their disease course, which may result in lead-time bias when comparing mortality between the 2 waves. Indeed, when 28-day mortality was investigated from the date of first symptoms rather than the date of presentation, the association between pandemic waves and mortality was attenuated in dialysis patients though not in kidney transplant recipients (Supplementary Tables S1–S4). These findings align with differences in health care utilization between dialysis patients and kidney transplant recipients, with dialysis patients requiring more frequent visits to health care facilities and therefore having a higher likelihood of being screened for COVID-19.S2

Among kidney transplant recipients, the younger age of patients in the second wave compared with the first wave also contributed to the lower crude mortality rate during the second wave. In our data, when explored further, age alone explained 43%, and the presence of limited or no symptoms together with age explained 61% of the lower risk of mortality in the second wave compared with the first wave among kidney transplant recipients. The reason for a younger average age among kidney transplant recipients with COVID-19 during the second wave could be related to the then available knowledge of a high risk of COVID-19 mortality in older people.S3^,^S4 In response, older kidney transplant recipients may have shielded themselves more stringently during the second wave whereas this was not possible in dialysis patients, who had to visit health care facilities regularly.S5^,^S6

Changes over time in the clinical management of kidney replacement therapy patients with COVID-19 were also observed. For example, fewer antiviral medications, more anti-inflammatory medications, and less adjustment of immunosuppressants (mainly in kidney transplant recipients) were used during the second wave compared with the first wave. This trend could be related to emerging evidence for the lack of a meaningful relationship between use of antiviral medications, and adjustment of immunosuppressants with mortality in individuals with COVID-19.S7–S12 Nevertheless, the lack of an association between pandemic wave and mortality, after accounting for disease severity, suggests that the increased identification of less severe cases was the main reason for lower risk of mortality during the second wave. An additional argument supporting this assumption is that there was no difference in mortality among patients who met the need for hospitalization in the 2 waves, and it can be assumed that the threshold for hospitalization did not change over time.

## Disclosure

All the authors declared no competing interests.

## Supplementary Material

**Supplementary Methods.**

**Supplementary Results.**

**Supplementary References.**

**Figure S1.** Daily reported COVID-19 cases of dialysis patients (panel A, *n* = 3004) and kidney transplant recipients (panel B, *n* = 1035) between March 1, 2020 and February 28, 2021

**Figure S2.** Kaplan-Meier curves for 28-day mortality by COVID-19 pandemic waves in the total population (left panels) and the population of hospitalized patients (right panels) for dialysis patients (upper panels) and kidney transplant recipients (lower panels)

**Figure S3.** Kaplan-Meier curves for 3-month mortality by COVID-19 pandemic waves in the total population (left panels) and the population of hospitalized patients (right panels) for dialysis patients (upper panels) and kidney transplant recipients (lower panels)

**Figure S4.** Kaplan-Meier curves for 28-day mortality by COVID-19 pandemic waves in the ICU admitted dialysis patients (left panel) and Intensive Care Unit admitted kidney transplant recipients (right panel)

**Figure S5.** Association between pandemic waves (second vs. first) and 28-day mortality in dialysis patients across key subgroups (Presented hazard ratios are from fully adjusted model∗)

**Figure S6.** Association between pandemic waves (second vs. first) and 28-day mortality in kidney transplant recipients across key subgroups

**Table S1.** Baseline patient and disease characteristics by COVID-19 pandemic waves in dialysis patients and kidney transplant recipients

**Table S2.** Twenty eight day mortality with follow-up starting at the first day of symptoms instead of at date of presentation in the second pandemic wave (vs. the first wave) in the population of dialysis patients (presented are hazard ratios with 95% confidence intervals)

**Table S3.** Twenty eight day mortality with follow-up starting at the first day of symptoms instead of at date of presentation in the second pandemic wave (vs. the first wave) in hospitalized dialysis patients only (presented are hazard ratios with 95% confidence intervals)

**Table S4.** Twenty eight day mortality with follow-up starting at the first day of symptoms instead of at date of presentation in the second pandemic wave (vs. the first wave) in the population of kidney transplant recipients (presented are hazard ratios with 95% confidence intervals)

**Table S5.** Twenty eight day mortality with follow-up starting at the first day of symptoms instead of at date of presentation in the second pandemic wave (vs. the first wave) in the population of hospitalized kidney transplant recipients (presented are hazard ratio with 95% confidence interval)

**Table S6.** Twenty eight day mortality in the second pandemic wave (vs. the first wave) among total and hospitalized dialysis patients when accounting for country effect (presented are hazard ratios with 95% confidence intervals)

**Table S7.** Twenty eight day mortality in the second pandemic wave (vs. the first wave) in total and hospitalized kidney transplant recipients when accounting for country effect (presented are hazard ratios with 95% confidence intervals)

**Table S8.** Twenty eight day mortality in the second pandemic wave (vs. the first wave) in ICU admitted dialysis patients (presented are hazard ratios with 95% confidence intervals)

**Table S9.** Twenty eight day mortality in the second pandemic wave (vs. the first wave) in ICU admitted kidney transplant recipients (presented are hazard ratios with 95% confidence intervals)

**Table S10.** Twenty eight day mortality in the second pandemic wave (vs. the first wave) in total and hospitalized dialysis patients when using country specific date for onset of second wave (presented are hazard ratios with 95% confidence intervals)

**Table S11.** Twenty eight day mortality in the second pandemic wave (vs. the first wave) in total and hospitalized kidney transplant recipients country specific date for onset of second wave (presented are hazard ratios with 95% confidence intervals)

**Table S12.** Twenty eight day mortality in the second pandemic wave (vs. the first wave) among total and hospitalized dialysis patients when considering end of the first wave on July 15, 2020 and the start of the second wave on August 15, 2020 (presented are hazard ratios with 95% confidence intervals)

**Table S13.** Twenty eight day mortality in the second pandemic wave (vs. the first wave) among total and hospitalized kidney transplant recipients when considering end of the first wave on July 15, 2020 and the start of the second wave on August 15, 2020 (presented are hazard ratios with 95% confidence intervals)

**Table S14.** Twenty eight day mortality in the second pandemic wave (vs. the first wave) in hospitalized and nonhospitalized dialysis patients by type of dialysis modality (presented are hazard ratios with 95% confidence intervals)

**Table S15.** Baseline patient and disease characteristics by COVID-19 pandemic waves in hospitalized dialysis patients (*N* = 1668). (First wave: From March 1, 2020 to July 31, 2020. Second wave: from August 1, 2020 to February 28, 2021).

**Table S16.** Baseline patient and disease characteristics by COVID-19 pandemic waves in hospitalized kidney transplant recipients (First wave: From March 1, 2020 to July 31, 2020. Second wave: from August 1, 2020 to February 28, 2021)

**STROBE statement.**
