# Supplementary material for: COVID-19 Pandemic Waves and Mortality Among Patients on Kidney Replacement Therapy
Source: Kidney Int Rep. 2022 Jun 20;7(9):2091–6. doi: 10.1016/j.ekir.2022.06.007 (PMC9213008; doi:10.1016/j.ekir.2022.06.007)
Supplement: Supplementary File (PDF) [file mmc1.pdf]

SUPPLEMENTARY MATERIAL

Contents

1) Methods.

2) Results.

3) Tables and Figures

Table S1: Baseline patient and disease characteristics by COVID-19 pandemic waves in dialysis patients and kidney transplant recipients

Table S2: 28-day mortality with follow-up starting at the first day of symptoms instead of at date of presentation in the second pandemic wave (versus the first wave) in the population of dialysis patients (presented are hazard ratios with 95% confidence intervals)

Table S3: 28-day mortality with follow-up starting at the first day of symptoms instead of at date of presentation in the second pandemic wave (versus the first wave) in hospitalized dialysis patients only (presented are hazard ratios with 95% confidence intervals)

Table S4: 28-day mortality with follow-up starting at the first day of symptoms instead of at date of presentation in the second pandemic wave (versus the first wave) in the population of kidney transplant recipients (presented are hazard ratios with 95% confidence intervals)

Table S5: 28-day mortality with follow-up starting at the first day of symptoms instead of at date of presentation in the second pandemic wave (versus the first wave) in the population of hospitalized kidney transplant recipients (presented are hazard ratio with 95% confidence interval)

Table S6: 28-day mortality in the second pandemic wave (versus the first wave) among total and hospitalized dialysis patients when accounting for country effect (presented are hazard ratios with 95% confidence intervals)

Table S7: 28-day mortality in the second pandemic wave (versus the first wave) in total and hospitalized kidney transplant recipients when accounting for country effect (presented are hazard ratios with 95% confidence intervals)

Table S8: 28-day mortality in the second pandemic wave (versus the first wave) in ICU admitted dialysis patients (presented are hazard ratios with 95% confidence intervals)

Table S9: 28-day mortality in the second pandemic wave (versus the first wave) in ICU admitted kidney transplant recipients (presented are hazard ratios with 95% confidence intervals)

Table S10: 28-day mortality in the second pandemic wave (versus the first wave) in total and hospitalized dialysis patients when using country specific date for onset of second wave (presented are hazard ratios with 95% confidence intervals)

Table S11: 28-day mortality in the second pandemic wave (versus the first wave) in total and hospitalized kidney transplant recipients country specific date for onset of second wave (presented are hazard ratios with 95% confidence intervals)

Table S12: 28-day mortality in the second pandemic wave (versus the first wave) among total and hospitalized dialysis patients when considering end of the first wave on July 15, 2020 and the start of the second wave on August 15, 2020 (presented are hazard ratios with 95% confidence intervals)

Table S13: 28-day mortality in the second pandemic wave (versus the first wave) among total and hospitalized kidney transplant recipients when considering end of the first wave on July 15, 2020 and the start of the second wave on August 15, 2020 (presented are hazard ratios with 95% confidence intervals)

Table S14: 28-day mortality in the second pandemic wave (versus the first wave) in hospitalized and non-hospitalized dialysis patients by type of dialysis modality (presented are hazard ratios with 95% confidence intervals)

Table S15: Baseline patient and disease characteristics by COVID-19 pandemic waves in hospitalized dialysis patients (N=1,668). (First wave: From March 1, 2020 to July 31, 2020. Second wave: from August 1, 2020 to February 28, 2021).

Table S16: Baseline patient and disease characteristics by COVID-19 pandemic waves in hospitalized kidney transplant recipients (First wave: From March 1, 2020 to July 31, 2020. Second wave: from August 1, 2020 to February 28, 2021)

Figure S1: Daily reported COVID-19 cases of dialysis patients (panel A, n=3,004) and kidney transplant recipients (panel B, n=1,035) between March 1st, 2020 and February 28th, 2021

Figure S2: Kaplan-Meier curves for 28-day mortality by COVID-19 pandemic waves in the total population (left panels) and the population of hospitalized patients (right panels) for dialysis patients (upper panels) and kidney transplant recipients (lower panels)

Figure S3: Kaplan-Meier curves for 3-month mortality by COVID-19 pandemic waves in the total population (left panels) and the population of hospitalized patients (right panels) for dialysis patients (upper panels) and kidney transplant recipients (lower panels)

Figure S4: Kaplan-Meier curves for 28-day mortality by COVID-19 pandemic waves in the ICU admitted dialysis patients (left panel) and ICU admitted kidney transplant recipients (right panel)

Figure S5: Association between pandemic waves (second vs. first) and 28-day mortality in dialysis patients across key subgroups (Presented hazard ratios are from fully adjusted model*)

Figure S6: Association between pandemic waves (second vs. first) and 28-day mortality in kidney transplant recipients across key subgroups

4) Additional references

5) STROBE statement

Supplementary material is linked to the online version of the paper at www.kireports.org

METHODS

Study design and participants

The ERACODA database was used as the data source for the present study.[1] This database was established in March 2020 to study prognosis and risk factors for mortality among kidney failure patients with COVID-19. Data were collected on adult (≥18 years) patients either on dialysis (hemodialysis or peritoneal dialysis) or living with a functioning kidney allograft, who were diagnosed with COVID-19 based on a positive result on a real-time polymerase chain reaction assay or rapid antigen test of nasal and/or pharyngeal swab specimens, and/or compatible findings on CT scan or chest X-ray of the lungs. The database currently involves the cooperation of approximately 225 physicians representing over 140 centers in 35 countries, mostly in Europe. Data were voluntarily reported on outpatients and hospitalized patients by physicians responsible for their care.

The ERACODA database is hosted at the University Medical Center Groningen, the Netherlands. Data is recorded using REDCap software (Research Electronic Data Capture, Vanderbilt University Medical Center, Nashville, TN, USA) for data collection.[2] Patient information is stored pseudonymized. The study was approved by the Institutional Review Board of the University Medical Center Groningen (Netherlands). Because of the observational, non-interventional nature, the institutional review board deemed the collection and analysis of data exempt from ethics review regarding the Dutch Medical Research Involving Human Subjects Act (WMO).

Data collection

Detailed information was collected on patient characteristics (age, sex, race/ethnicity, height, weight, frailty, comorbidities, hospitalization, and medication use) and COVID-19 related characteristics (reason for COVID-19 screening, symptoms, vital signs, and laboratory test results) at presentation. Frailty was assessed using the Clinical Frailty Score developed by Rockwood et al..[3] Data was also collected and

reported on eGFR, C-reactive protein and lymphocytes count which are known prognostic markers among individuals with COVID-19.[4-7] For the analysis, all patients who presented between March 1st, 2020 and February 28th, 2021 and had information on the date of presentation, type of renal replacement therapy, and 28-day mortality were included. The first wave of the pandemic was assumed to end on July 31st, 2020, based on the lowest number of daily reported cases in this period before an increase in reported cases thereafter. Patients in the second wave were included until February 28th, 2021 because after this period COVID-19 vaccination was available for kidney patients in most European countries and new COVID-19 variants became dominant, which both affected mortality rates.[8,9] Total 28-day mortality was chosen as a primary outcome. The crude mortality rate was defined as the percentage of patients who died within 28 days of presentation (numerator) among those who presented with COVID-19 (denominator). To explore potential reasons for any difference in mortality between the two waves, the percentages of patients with limited to no symptoms (defined as patients not experiencing any of the recorded symptoms including sore throat, cough, shortness of breath, fever, headache, nausea or vomiting, diarrhea, and myalgia or arthralgia), patients identified through routine screening, and hospitalization were investigated. Additionally, assuming that there were no differences in disease severity to warrant hospitalization during the first and the second wave, we also investigated mortality among hospitalized and non-hospitalized patients separately to account for the possibility of greater identification of asymptomatic, or less severe cases in the second wave.

Statistical analysis

Baseline characteristics were presented by pandemic wave (first/second) for dialysis patients and kidney transplant recipients, separately. Dialysis patients and kidney transplant recipients were analyzed separately due to differences in their clinical characteristics and management. Continuous data were presented as mean (standard deviation (SD)) or as median (interquartile interval (IQI)) in case of a non-

Gaussian distribution of data. Categorical data were presented as numbers (percentages). Baseline characteristics were compared between the first and the second wave using independent sample t-test (in case of Gaussian distribution) or the Mann Whitney U-test (in case of non-Gaussian distribution) for continuous variables and Pearson Chi-2 test for categorical variables. To identify differences that are not only statistically significant but likely also relevant, the standardized mean difference (SMD) in baseline characteristics between two waves for both continuous and categorical variables was also calculated. SMD-estimates are based only on sample statistics and are not directly influenced by sample size.[10] An SMD of 0.15 or more was used to indicate a relevant difference in baseline characteristics between the two waves.[11] A complete case-analysis was performed.

Percentages of patients experiencing 28-day mortality, and in-hospital mortality were compared between the two waves and corresponding p-values were calculated using the Pearson Chi-2 test. Further, hazard ratios (HRs) and 95% confidence intervals (CIs) for the association of pandemic waves (second versus first (reference)) with total 28-day mortality and in-hospital mortality were estimated using Cox proportional-hazards regression models. To understand the relationship of pandemic waves with 28-day mortality and to examine factors that may explain any observed difference in 28-day mortality between the two waves, multiple models were constructed and relevant factors were added in a stepwise manner. Model 1 was a crude (unadjusted) model. In Model 2 we adjusted for patient demographics i.e. age (continuous) and sex (male/female). In model 3 we additionally adjusted for reasons for COVID-19 screening (symptoms only/symptoms & COVID-19+ contact/COVID-19+ contact only/routine) and presence of disease symptoms (yes/no). Model 4 was further adjusted for factors known to be associated with COVID-19 outcome, i.e. smoking (never, current, former), obesity (yes/no), hypertension (yes/no), diabetes (yes/no), heart failure (yes/no), chronic lung disease (yes/no), coronary artery disease (yes/no), auto-immune disease (yes/no), malignancy (yes/no) and clinical frailty score (continuous). In model 5, we additionally adjusted for disease severity related factors including cough

(yes/no), shortness of breath (yes/no), fever (yes/no), sore throat (yes/no), O2 saturation (continuous), pulse (continuous), temperature (continuous), systolic blood pressure (continuous), diastolic blood pressure (continuous), lymphocytes (continuous), C-reactive protein (CRP) (log, continuous). In the final model (Model 6), we further adjusted for hospitalization (yes/no) as an additional marker of disease severity, except when investigating in-hospital mortality. The proportional-hazards assumption was investigated by testing the interaction of log(time) with individual covariates. Kaplan-Meier curves were plotted to show cumulative 28-day survival by the pandemic wave. The log-rank test was used to compare cumulative survival between two waves.

We performed several additional analyses. First, to assess lead-time bias, we investigated the relationship between pandemic wave and mortality when starting follow-up from the date of first symptoms instead of date of diagnosis. Second, we constructed a random intercept model with country as a random factor in a multilevel mixed-effects parametric survival model. Third, to investigate potential differences in mortality beyond 28-days, we examined the association of the pandemic waves with 3-month mortality. Fourth, to examine mortality in more severe cases, we investigated the difference in mortality between the two waves in ICU admitted patients. Fifth, to assess the robustness of our results, we used a country-specific cut-off date for the distinction between the first and the second wave since the onset of the first and second waves may differ across countries. Furthermore, to reduce the likelihood of overlap between the first and the second wave between countries, we repeated analyses when considering the end of first wave on July 15th 2020 and the start of second wave on August 15th, 2020. Sixth, we assessed consistency of our results across key subgroups including age (≤65/>65 years), sex (male/female), hypertension (no/yes) and diabetes (no/yes). Seventh, we examined the relationship between pandemic wave and mortality by type of dialysis modality. Finally, we compared patient characteristics between patients from the first and the second wave among hospitalized patients.

All analyses were performed using Stata version 17.0 (College Station, TX). A 2-sided p-value less than 0.05 was adopted to indicate statistical significance.

References

1) Noordzij M, Duivenvoorden R, Pena MJ, et al.: ERACODA: the European database collecting clinical information of patients on kidney replacement therapy with COVID-19. Nephrol Dial Transplant 35: 2023–2025, 2020
2) Harris PA, Taylor R, Minor BL, et al.: The REDCap consortium: Building an international community of software partners. J Biomed Inform 95: 103208, 2019
3) Rockwood K, Song X, MacKnight C et al. A global clinical measure of fitness and frailty in elderly people. CanMed Assoc J 2005; 173: 489–495
4) Carter B, Ramsay EA, Short R, Goodison S, Lumsden J, Khan A, Braude P, Vilches-Moraga A, Quinn TJ, McCarthy K, Hewitt J, Myint PK; COPE Study. Prognostic value of estimated glomerular filtration rate in hospitalised older patients (over 65) with COVID-19: a multicentre, European, observational cohort study. BMC Geriatr. 2022 Feb 12;22(1):119.
5) Stringer D, Braude P, Myint PK, Evans L, Collins JT, Verduri A, Quinn TJ, Vilches-Moraga A, Stechman MJ, Pearce L, Moug S, McCarthy K, Hewitt J, Carter B; COPE Study Collaborators. The role of C-reactive protein as a prognostic marker in COVID-19. Int J Epidemiol. 2021 May 17;50(2):420-429.
6) Illg Z, Muller G, Mueller M, Nippert J, Allen B. Analysis of absolute lymphocyte count in patients with COVID-19. Am J Emerg Med. 2021 Aug;46:16-19.
7) Wagner J, DuPont A, Larson S, Cash B, Farooq A. Absolute lymphocyte count is a prognostic marker in Covid-19: A retrospective cohort review. Int J Lab Hematol. 2020 Dec;42(6):761-765.
8) https://ec.europa.eu/info/live-work-travel-eu/coronavirus-response/public-health/eu-vaccines-strategy_en#objectives-of-the-strategy. Accessed on September 15, 2021
9) https://www.ecdc.europa.eu/en/covid-19/variants-concern. Accessed on September 15, 2021
10) Nguyen TL, Xie L. Incomparability of treatment groups is often blindly ignored in randomised controlled trials - a post hoc analysis of baseline characteristic tables. J Clin Epidemiol. 2021 Feb;130:161-168.
11) Lovakov A, Agadullina ER. Empirically Derived Guidelines for Effect Size Interpretation in Social Psychology. European Journal of Social Psychology. 2021; 3: 485-504

Results

Among dialysis patients, anti-viral medication use was lower, whereas anti-inflammatory medication use was higher in the second wave. Cough, shortness of breath, and fever were substantially less common in the second wave. Patients in the second wave had a lower body temperature, respiration rate, and pulse rate.

Kidney transplant recipients in the second wave were tested more frequently because of a COVID-19 positive contact without having symptoms themselves. As in dialysis patients, the use of anti-viral medications was lower and the use of anti-inflammatory medications higher in the second wave. In addition, adjustment of immunosuppressive therapy after presentation was less common in the second wave. The prevalence of disease-related symptoms including cough, and fever was generally lower, while only the prevalence of sore throat and headache was higher in the second wave (Table S1).

Additional analyses

When investigating the risk of 28-day mortality from the date of first symptoms instead of the date of presentation in dialysis patients to account for possible lead-time bias, further attenuation of the association of the second wave with lower mortality was observed in all models, both in the overall population and in hospitalized patients only (Tables S2 and S3). In kidney transplant recipients, similar results were obtained by using either the date of first symptoms or the date of presentation as the start of follow-up (Tables S4 and S5). Results were essentially similar when accounting for between-country differences in the relationship between pandemic wave (Tables S6 and S7). During the 3-month follow-up since the presentation, a total of 354 (28.2%) patients died during the first wave and 404 (21.3%) patients during the second wave in dialysis patients (p for difference=0.001). The corresponding numbers in kidney transplant recipients were 101 (21.3%) and 90 (16.1%) (p-for difference=0.04). Kaplan-Meier curves for 3-month mortality were essentially similar to the results for 28-day mortality in

the overall populations of dialysis and kidney transplant recipients (Figure S3). Among hospitalized dialysis patients, three months after presentation 318 (35.6%) had died during the first wave and 272 (35.1%) during the second wave (p=0.83). Among hospitalized kidney transplant recipients, these data were 98 (24.6%) and 88 (25.7%) (p=0.71), respectively. Kaplan-Meier curves for 3-month mortality demonstrated no difference in cumulative survival between the two waves in hospitalized patients (p=0.84 in dialysis patients and p=0.76 in transplant recipients) with even an indication of lower cumulative survival after the first month after the presentation in the second wave (Figure S3). No heterogeneity was observed in rates of mortality in ICU admitted patients in the two pandemic waves both in dialysis patients and kidney transplant recipients (Tables S8 and S9; Figures S4). Results were essentially similar when using country-specific cut-off dates to define the onset of the second wave (Tables S10 and S11) and when allowing a month's gap between the end of the first wave and the start of the second wave (Table S12 and S13). Finally, our main finding were consistent across key subgroups (Figures S5 and S6) and when investigated by type of dialysis modality (p for interaction=0.46 in total dialysis population; p for interaction=0.67 in hospitalized dialysis population) (Table S14). Characteristics of hospitalized patients in the first and the second wave were largely comparable (Tables S15 and S16).

Table S1: Baseline patient and disease characteristics by COVID-19 pandemic waves in dialysis patients and kidney transplant recipients

	Dialysis patients (N=3,004)				Kidney transplant recipients (N=1,035)			
	Pandemic waves				Pandemic waves			
	First wave (N=1,253)	Second wave (N=1,751)	p-value	Std. difference	First wave (N=475)	Second wave (N=560)	p-value	Std. difference
**Patient characteristics**								
Female, n (%)	494 (39.4)	646 (36.9)	0.16	0.05	184 (38.7)	215 (38.4)	0.91	0.01
Age, (years)	66.5 (14.5)	68.7 (14.1)	<0.001	-0.16	58.2 (13.5)	55.6 (13.6)	0.002	0.19
Body Mass Index, (kg/m^2)	26.6 (5.6)	26.8 (5.4)	0.39	-0.03	27.0 (4.9)	27.2 (5.2)	0.56	-0.04
Caucasians, n (%)	1066 (87.0)	1396 (85.0)	0.12	0.06	392 (86.5)	453 (82.8)	0.11	0.10
Tobacco use, n (%)			<0.001	0.33			0.52	0.09
Current	86 (6.9)	100 (5.7)			18 (3.8)	24 (4.3)		
Prior	259 (20.7)	316 (18.0)			111 (23.4)	116 (20.7)		
Never	525 (41.9)	530 (30.3)			243 (51.2)	280 (50.0)		
Unknown	383 (30.6)	805 (46.0)			103 (21.7)	140 (25.0)		
Reason for screening*, n(%)			<0.001	0.24			<0.001	0.43
Symptoms only	664 (67.1)	765 (59.9)			319 (89.4)	413 (74.1)		
Symptoms & COVID+ Contact	159 (16.1)	172 (13.5)			24 (6.7)	88 (15.8)		
COVID+ Contact only	82 (8.3)	172 (13.5)			4 (1.1)	37 (6.6)		
Routine	85 (8.6)	169 (13.2)			10 (2.8)	19 (3.4)		
Clinical frailty scale, AU	4.0 (1.8)	4.0 (1.8)	0.95	0.00	3.0 (1.6)	2.9 (1.4)	0.65	0.07
Comorbidities, n (%)								
Hypertension	1049 (83.7)	1369 (78.2)	<0.001	0.14	397 (83.6)	449 (80.2)	0.16	0.09
Diabetes Mellitus	533 (42.5)	737 (42.1)	0.81	0.01	141 (29.7)	181 (32.3)	0.36	-0.06
Coronary artery disease	418 (33.4)	606 (34.6)	0.48	-0.03	92 (19.4)	91 (16.3)	0.19	0.08
Heart failure	294 (23.5)	410 (23.4)	0.97	0.00	39 (8.2)	57 (10.2)	0.28	-0.07
Chronic lung disease	180 (14.4)	205 (11.7)	0.03	0.08	37 (7.8)	41 (7.3)	0.78	0.02
Active malignancy	83 (6.6)	85 (4.9)	0.04	0.08	25 (5.3)	17 (3.0)	0.07	0.11
Auto-immune disease,	53 (4.2)	61 (3.5)	0.29	0.04	24 (5.1)	33 (5.9)	0.55	-0.04
Primary kidney disease, n (%)								
Primary Glomerulonephritis	180 (14.7)	166 (10.1)	<0.001	0.14	90 (19.5)	91 (16.3)	0.19	0.08

Pyelonephritis	20 (1.6)	26 (1.6)	0.93	0.00	17 (3.7)	11 (2.0)	0.10	0.10
Interstitial nephritis	42 (3.4)	37 (2.3)	0.06	0.07	20 (4.3)	18 (3.2)	0.35	0.06
Hereditary kidney disease	91 (7.4)	99 (6.0)	0.14	0.05	64 (13.9)	72 (12.9)	0.65	0.03
Congenital diseases	18 (1.5)	23 (1.4)	0.89	0.01	16 (3.5)	29 (5.2)	0.18	-0.09
Vascular diseases	166 (13.5)	368 (22.5)	<0.001	-0.23	36 (7.8)	45 (8.1)	0.87	-0.01
Sec. glomerular disease	98 (8.0)	147 (9.0)	0.35	-0.04	21 (4.6)	31 (5.6)	0.47	-0.05
Diabetic kidney disease	287 (23.4)	350 (21.4)	0.20	0.05	57 (12.4)	55 (9.9)	0.21	0.08
Other	224 (18.2)	308 (18.8)	0.71	-0.01	72 (15.6)	131 (23.5)	0.002	-0.20
Unknown	102 (8.3)	115 (7.0)	0.20	0.05	68 (14.8)	74 (13.3)	0.50	0.04
Dialysis duration, years	5 (3, 8)	4 (2, 7)	0.001	0.09				
Transplant duration, n (%)							0.007	0.16
<1 year	-	-	-	-	30 (6.4)	67 (12.0)		
1-5 years	-	-	-	-	182 (38.6)	211 (37.9)		
>5 years	-	-	-	-	260 (55.1)	278 (50.0)		
**Medications[ᵼ], n (%)**								
Immunosuppressants							0.02	0.18
Monotherapy	-	-	-	-	18 (3.8)	8 (1.4)		
Dual therapy	-	-	-	-	150 (32.1)	208 (37.5)		
Triple therapy	-	-	-	-	300 (64.1)	339 (61.1)		
Immunosuppressants adjustment[α]	-	-	-	-	344 (72.4)	251 (44.8)	<0.001	0.58
Start of anti-viral drugs	537 (62.1)	161 (20.8)	<0.001	0.92	279 (59.1)	107 (19.2)	<0.001	0.84
Start of anti-inflammatory drugs	125 (14.4)	363 (46.9)	<0.001	-0.75	96 (20.3)	196 (35.2)	<0.001	-0.71
**Disease characteristics**								
Presenting symptoms, n (%)								
Sore throat	130 (10.4)	177 (13.8)	0.04	-0.09	63 (13.3)	104 (18.6)	0.005	0.18
Cough	588 (52.0)	556 (44.6)	<0.001	0.15	305 (65.2)	296 (56.1)	0.003	0.19
Shortness of breath	408 (36.0)	353 (28.3)	<0.001	0.17	202 (43.3)	199 (37.5)	0.06	0.12
Fever	714 (62.4)	562 (45.3)	<0.001	0.35	342 (73.2)	338 (63.1)	0.001	0.22
Headache	117 (9.4)	117 (9.2)	0.60	0.02	75 (15.9)	123 (22.0)	0.003	0.20
Nausea or vomiting	157 (12.6)	113 (8.8)	0.56	0.02	73 (15.5)	78 (14.0)	0.85	0.01
Diarrhoea	120 (9.6)	139 (10.9)	0.48	-0.03	127 (27.0)	133 (23.8)	0.60	0.03
Myalgia or arthralgia	228 (18.3)	270 (21.2)	0.21	-0.05	130 (27.6)	161 (28.8)	0.39	-0.06
No to limited symptoms	164 (14.3)	312 (24.9)	<0.001	0.27	10 (2.1)	46 (8.5)	<0.001	-0.29

Vital signs								
Temperature, °C	37.5 (1.0)	37.3 (1.0)	<0.001	0.17	37.5 (1.1)	37.6 (1.0)	0.45	-0.05
Respiration rate, /min	19 (5)	18 (5)	<0.001	0.36	21 (7)	20 (7)	0.03	0.16
O2 saturation room air, %	93.7 (5.1)	94.2 (5.8)	0.04	-0.09	93.7 (7.8)	93.8 (8.9)	0.84	-0.01
Systolic BP, mm Hg	136.3 (25.7)	136.5 (25.2)	0.86	-0.01	131.5 (20.4)	133.9 (21.5)	0.14	-0.11
Diastolic BP, mm Hg	74.5 (15.3)	72.5 (15.3)	0.01	0.13	77.0 (13.6)	79.6 (14.5)	0.02	-0.19
Pulse rate, BPM	82 (15)	80 (15)	0.01	0.13	86 (17)	89 (17)	0.01	-0.20
Laboratory test results								
eGFR, ml/min/1.73m^2	-	-	-	-	40.6 (23.6)	44.5 (36.4)	0.06	-0.13
Lymphocytes, x1000/µL	0.9 (0.6, 1.3)	0.9 (0.6, 1.3)	0.74	0.01	0.8 (0.5, 1.3)	0.8 (0.5, 1.4)	0.89	-0.04
CRP, mg/L	25 (6, 74)	28 (8, 83)	0.06	-0.08	50 (12, 99)	33 (7, 87)	0.005	0.17

Continuous variables are reported as mean (standard deviation) or median (Inter quartile interval). Groups were compared using independent sample t-test, Mann-Whitney U-test, or Pearson Chi-square test as appropriate. Obesity is defined as BMI >30 kg/m^2. *Abbreviations are:* Std., standardized; °C, degree Celsius; O2, oxygen; BP, blood pressure; BPM, beats per minute; eGFR, estimated glomerular filtration rate; CRP, C-reactive protein;

*Total number may not add up due to missing data; [ⱡ]In hospitalized patients only, [α]within 48 hours of presentation

Table S2: 28-day mortality with follow-up starting at the first day of symptoms instead of at date of presentation in the second pandemic wave (versus the first wave) in the population of dialysis patients (presented are hazard ratios with 95% confidence intervals)

	Pandemic waves (N=2,221)		
	**First wave (N=1,073)**	**Second wave (N=1,148)**	**p-value**
**Mortality, n (%)**	**243 (22.7)**	**344 (20.1)**	
Model 1	Ref.	0.90 (0.75, 1.07)	0.23
Model 2	Ref.	0.82 (0.69, 0.98)	0.03
Model 3	Ref.	0.89 (0.75, 1.07)	0.22
Model 4	Ref.	0.92 (0.76, 1.10)	0.37
Model 5	Ref.	0.93 (0.77, 1.13)	0.46
Model 6	Ref.	0.98 (0.81, 1.18)	0.83

Model 1: crude
Model 2: Model 1 + age, and sex
Model 3: Model 2 + reason for screening, and presence of no to limited symptoms
Model 4: Model 3 + smoking, hypertension, diabetes mellitus, chronic lung disease, heart failure, chronic artery disease, auto-immune disease, malignancy, and frailty score
Model 5: Model 4 + cough, shortness of breath, fever, sore throat, O2 saturation, pulse, temperature, systolic blood pressure, diastolic blood pressure, lymphocytes, and c-reactive protein
Model 6: Model 5 + hospitalization

Table S3: 28-day mortality with follow-up starting at the first day of symptoms instead of at date of presentation in the second pandemic wave (versus the first wave) in hospitalized dialysis patients only (presented are hazard ratios with 95% confidence intervals)

	Pandemic waves (N=1,494)		
	**First wave (N=802)**	**Second wave (N=692)**	**p-value**
**Mortality, n (%)**	**219 (27.3)**	**217 (31.4)**	
Model 1	Ref.	1.13 (0.94, 1.36)	0.20
Model 2	Ref.	1.02 (0.84, 1.23)	0.87
Model 3	Ref.	1.05 (0.87, 1.27)	0.63
Model 4	Ref.	1.05 (0.87, 1.28)	0.59
Model 5	Ref.	1.03 (0.85, 1.26)	0.75

Model 1: crude
Model 2: Model 1 + age, and sex
Model 3: Model 2 + reason for screening, and presence of no to limited symptoms
Model 4: Model 3 + smoking, hypertension, diabetes mellitus, chronic lung disease, heart failure, chronic artery disease, auto-immune disease, malignancy, and frailty score
Model 5: Model 4 + cough, shortness of breath, fever, sore throat, O2 saturation, pulse, temperature, systolic blood pressure, diastolic blood pressure, lymphocytes, and c-reactive protein

Table S4: 28-day mortality with follow-up starting at the first day of symptoms instead of at date of presentation in the second pandemic wave (versus the first wave) in the population of kidney transplant recipients (presented are hazard ratios with 95% confidence intervals)

	Pandemic waves (N=917)		
	**First wave (N=414)**	**Second wave (N=503)**	**p-value**
**Mortality, n (%)**	**73 (17.6)**	**60 (11.9)**	
Model 1	Ref.	0.66 (0.47, 0.93)	0.02
Model 2	Ref.	0.76 (0.54, 1.07)	0.11
Model 3	Ref.	0.80 (0.56, 1.13)	0.21
Model 4	Ref.	0.82 (0.57, 1.16)	0.26
Model 5	Ref.	0.87 (0.60, 1.28)	0.48
Model 6	Ref.	1.02 (0.70, 1.49)	0.91

Model 1: crude
Model 2: Model 1 + age, and sex
Model 3: Model 2 + reason for screening, and presence of no to limited symptoms
Model 4: Model 3 + smoking, hypertension, diabetes mellitus, chronic lung disease, heart failure, chronic artery disease, auto-immune disease, malignancy, and frailty score
Model 5: Model 4 + cough, shortness of breath, fever, sore throat, O2 saturation, pulse, temperature, systolic blood pressure, diastolic blood pressure, lymphocytes, and c-reactive protein
Model 6: Model 5 + hospitalization

Table S5: 28-day mortality with follow-up starting at the first day of symptoms instead of at date of presentation in the second pandemic wave (versus the first wave) in the population of hospitalized kidney transplant recipients (presented are hazard ratio with 95% confidence interval)

	Pandemic waves (N=664)		
	**First wave (N=354)**	**Second wave (N=310)**	**p-value**
**Mortality, n (%)**	**71 (20.1)**	**58 (18.7)**	
Model 1	Ref.	0.93 (0.66, 1.32)	0.68
Model 2	Ref.	0.95 (0.67, 1.35)	0.78
Model 3	Ref.	0.97 (0.68, 1.37)	0.84
Model 4	Ref.	1.01 (0.70, 1.45)	0.97
Model 5	Ref.	1.06 (0.72, 1.54)	0.78

Model 1: crude
Model 2: Model 1 + age, and sex
Model 3: Model 2 + reason for screening, and presence of no to limited symptoms
Model 4: Model 3 + smoking, hypertension, diabetes mellitus, chronic lung disease, heart failure, chronic artery disease, auto-immune disease, malignancy, and frailty score
Model 5: Model 4 + cough, shortness of breath, fever, sore throat, O2 saturation, pulse, temperature, systolic blood pressure, diastolic blood pressure, lymphocytes, and c-reactive protein

Table S6: 28-day mortality in the second pandemic wave (versus the first wave) among total and hospitalized dialysis patients when accounting for country effect (presented are hazard ratios with 95% confidence intervals)

**Total**	**Pandemic waves (N=3,004)**		
	**First wave (N=1,253)**	**Second wave (N=1,751)**	**p-value**
**Mortality, n (%)**	**304 (24.3)**	**344 (19.6)**	
Model 1	Ref.	0.73 (0.62, 0.87)	<0.001
Model 2	Ref.	0.69 (0.59, 0.82)	<0.001
Model 3	Ref.	0.75 (0.64, 0.89)	0.001
Model 4	Ref.	0.78 (0.66, 0.92)	0.003
Model 5	Ref.	0.80 (0.67, 0.94)	0.009
Model 6	Ref.	0.90 (0.75, 1.07)	0.23
**Hospitalized**	**Pandemic waves (N=1,668)**		
	**First wave (N=893)**	**Second wave (N=775)**	**p-value**
**Mortality, n (%)**	**274 (30.7)**	**236 (30.4)**	
Model 1	Ref.	0.82 (0.68, 1.00)	0.05
Model 2	Ref.	0.84 (0.69, 1.02)	0.07
Model 3	Ref.	0.86 (0.71, 1.04)	0.12
Model 4	Ref.	0.85 (0.70, 1.02)	0.09
Model 5	Ref.	0.86 (0.71, 1.05)	0.14

Model 1: crude
Model 2: Model 1 + age, and sex
Model 3: Model 2 + reason for screening, and presence of no to limited symptoms
Model 4: Model 3 + smoking, hypertension, diabetes mellitus, chronic lung disease, heart failure, chronic artery disease, auto-immune disease, malignancy, and frailty score
Model 5: Model 4 + cough, shortness of breath, fever, sore throat, O2 saturation, pulse, temperature, systolic blood pressure, diastolic blood pressure, lymphocytes, and c-reactive protein
Model 6: Model 5 + hospitalization

Table S7: 28-day mortality in the second pandemic wave (versus the first wave) in total and hospitalized kidney transplant recipients when accounting for country effect (presented are hazard ratios with 95% confidence intervals)

Total	Pandemic waves (N=1,035)		
	First wave (N=475)	Second wave (N=560)	p-value
**Mortality, n (%)**	**89 (18.7)**	**72 (12.9)**	
Model 1	Ref.	0.66 (0.48, 0.91)	0.01
Model 2	Ref.	0.79 (0.58, 1.08)	0.14
Model 3	Ref.	0.85 (0.62, 1.17)	0.32
Model 4	Ref.	0.82 (0.60, 1.13)	0.23
Model 5	Ref.	0.83 (0.59, 1.17)	0.29
Model 6	Ref.	0.97 (0.69, 1.36)	0.84
**Hospitalized**	**Pandemic waves (N=741)**		
	**First wave (N=399)**	**Second wave (N=342)**	**p-value**
**Mortality, n (%)**	**87 (21.8)**	**70 (20.5)**	
Model 1	Ref.	0.91 (0.66, 1.26)	0.58
Model 2	Ref.	0.96 (0.70, 1.33)	0.81
Model 3	Ref.	1.00 (0.72, 1.38)	0.98
Model 4	Ref.	0.99 (0.72, 1.37)	0.97
Model 5	Ref.	0.99 (0.70, 1.39)	0.94

Model 1: crude
Model 2: Model 1 + age, and sex
Model 3: Model 2 + reason for screening, and presence of no to limited symptoms
Model 4: Model 3 + smoking, hypertension, diabetes mellitus, chronic lung disease, heart failure, chronic artery disease, auto-immune disease, malignancy, and frailty score
Model 5: Model 4 + cough, shortness of breath, fever, sore throat, O2 saturation, pulse, temperature, systolic blood pressure, diastolic blood pressure, lymphocytes, and c-reactive protein
Model 6: Model 5 + hospitalization

Table S8: 28-day mortality in the second pandemic wave (versus the first wave) in ICU admitted dialysis patients (presented are hazard ratios with 95% confidence intervals)

	Pandemic waves (N=231)		
	**First wave (N=122)**	**Second wave (N=109)**	**p-value**
**Mortality, n (%)**	**67 (54.9)**	**52 (47.7)**	
Model 1	Ref.	0.74 (0.52, 1.07)	0.11
Model 2	Ref.	0.71 (0.49, 1.02)	0.07
Model 3	Ref.	0.76 (0.52, 1.10)	0.14
Model 4	Ref.	0.74 (0.49, 1.10)	0.14
Model 5	Ref.	0.76 (0.49, 1.18)	0.21

Model 1: crude
Model 2: Model 1 + age, and sex
Model 3: Model 2 + reason for screening, and presence of no to limited symptoms
Model 4: Model 3 + smoking, hypertension, diabetes mellitus, chronic lung disease, heart failure, chronic artery disease, auto-immune disease, malignancy, and frailty score
Model 5: Model 4 + cough, shortness of breath, fever, sore throat, O2 saturation, pulse, temperature, systolic blood pressure, diastolic blood pressure, lymphocytes, and c-reactive protein

Table S9: 28-day mortality in the second pandemic wave (versus the first wave) in ICU admitted kidney transplant recipients (presented are hazard ratios with 95% confidence intervals)

	Pandemic waves (N=166)		
	**First wave (N=81)**	**Second wave (N=85)**	**p-value**
**Mortality, n (%)**	**34 (41.9)**	**33 (38.8)**	
Model 1	Ref.	0.90 (0.56, 1.45)	0.66
Model 2	Ref.	0.79 (0.48, 1.29)	0.35
Model 3	Ref.	0.78 (0.48, 1.29)	0.34
Model 4	Ref.	0.74 (0.43, 1.28)	0.28
Model 5	Ref.	0.80 (0.47, 1.30)	0.39

Model 1: crude
Model 2: Model 1 + age, and sex
Model 3: Model 2 + reason for screening, and presence of no to limited symptoms
Model 4: Model 3 + smoking, hypertension, diabetes mellitus, chronic lung disease, heart failure, chronic artery disease, auto-immune disease, malignancy, and frailty score
Model 5: Model 4 + cough, shortness of breath, fever, sore throat, O2 saturation, pulse, temperature, systolic blood pressure, diastolic blood pressure, lymphocytes, and c-reactive protein

Table S10: 28-day mortality in the second pandemic wave (versus the first wave) in total and hospitalized dialysis patients when using country specific date for onset of second wave (presented are hazard ratios with 95% confidence intervals)

**Total (N=3,004)**	**First wave (N=1,223)**	**Second wave (N=1,781)**	**p-value**
**Mortality, n (%)**	**298 (24.4)**	**350 (19.6)**	
Model 1	Ref.	0.76 (0.65, 0.89)	0.001
Model 2	Ref.	0.69 (0.59, 0.81)	<0.001
Model 3	Ref.	0.74 (0.64, 0.87)	<0.001
Model 4	Ref.	0.77 (0.66, 0.91)	0.001
Model 5	Ref.	0.79 (0.67, 0.92)	0.004
Model 6	Ref.	0.93 (0.79, 1.10)	0.40
**Hospitalized (N=1,668)**	**First wave (N=864)**	**Second wave (N=804)**	**p-value**
**Mortality, n (%)**	**268 (31.0)**	**242 (30.1)**	
Model 1	Ref.	0.92 (0.77, 1.09)	0.35
Model 2	Ref.	0.86 (0.72, 1.02)	0.08
Model 3	Ref.	0.88 (0.74, 1.05)	0.16
Model 4	Ref.	0.89 (0.74, 1.06)	0.19
Model 5	Ref.	0.89 (0.74, 1.06)	0.19

Model 1: crude
Model 2: Model 1 + age, and sex
Model 3: Model 2 + reason for screening, and presence of no to limited symptoms
Model 4: Model 3 + smoking, hypertension, diabetes mellitus, chronic lung disease, heart failure, chronic artery disease, auto-immune disease, malignancy, and frailty score
Model 5: Model 4 + cough, shortness of breath, fever, sore throat, O2 saturation, pulse, temperature, systolic blood pressure, diastolic blood pressure, lymphocytes, and c-reactive protein
Model 6: Model 5 + hospitalization

Country specific onset of second wave dates (selected a date closest to the first date of a month where the lowest number of daily cases were observed after first peak between March 1, 2021 and February 28, 2021): Albania June 1, 2020; Belgium August 1, 2020, Bosnia June 1, 2020; Croatia June 1, 2020; Czech Republic June 1, 2020; France July 1, 2020; Germany August 1, 2020; Greece August 1, 2020; Italy August 1, 2020; Lithuania August 1, 2020; Luxembourg June 1, 2020; Netherlands August 1, 2020; Norway August 1, 2020; Poland July 1, 2020; Portugal August 1, 2020; Romania June 1, 2020; Russia September 1, 2020; Serbia October 1, 2020; Slovakia August 1, 2020; Slovenia August 1, 2020; Spain July 1, 2020; Sweden September 1, 2020; Switzerland August 1, 2020; Ukraine June 1, 2020; United Kingdom August 1, 2020; Turkey August 1, 2020; Egypt October 1, 2020; Libya June 1, 2020; Morocco July 1, 2020; Syria June 1, 2020

Table S11: 28-day mortality in the second pandemic wave (versus the first wave) in total and hospitalized kidney transplant recipients country specific date for onset of second wave (presented are hazard ratios with 95% confidence intervals)

Total (N=1,035)	First wave (N=457)	Second wave (N=578)	p-value
**Mortality, n (%)**	**88 (19.3)**	**73 (12.6)**	
Model 1	Ref.	0.63 (0.46, 0.85)	0.003
Model 2	Ref.	0.78 (0.57, 1.06)	0.11
Model 3	Ref.	0.83 (0.61, 1.15)	0.26
Model 4	Ref.	0.80 (0.58, 1.11)	0.18
Model 5	Ref.	0.81 (0.58, 1.14)	0.23
Model 6	Ref.	0.94 (0.67, 1.31)	0.71
**Hospitalized (N=733)**	**First wave (N=386)**	**Second wave (N=347)**	**p-value**
**Mortality, n (%)**	**86 (22.3)**	**70 (20.2)**	
Model 1	Ref.	0.87 (0.64, 1.20)	0.40
Model 2	Ref.	0.95 (0.70, 1.31)	0.77
Model 3	Ref.	0.98 (0.72, 1.35)	0.92
Model 4	Ref.	0.96 (0.70, 1.33)	0.82
Model 5	Ref.	0.96 (0.68, 1.34)	0.80

Model 1: crude
Model 2: Model 1 + age, and sex
Model 3: Model 2 + reason for screening, and presence of no to limited symptoms
Model 4: Model 3 + smoking, hypertension, diabetes mellitus, chronic lung disease, heart failure, chronic artery disease, auto-immune disease, malignancy, and frailty score
Model 5: Model 4 + cough, shortness of breath, fever, sore throat, O2 saturation, pulse, temperature, systolic blood pressure, diastolic blood pressure, lymphocytes, and c-reactive protein
Model 6: Model 5 + hospitalization

Country specific onset of second wave dates: Albania June 1, 2020; Belgium August 1, 2020, Bosnia June 1, 2020; Croatia June 1, 2020; Czech Republic June 1, 2020; France July 1, 2020; Germany August 1, 2020; Greece August 1, 2020; Italy August 1, 2020; Lithuania August 1, 2020; Luxembourg June 1, 2020; Netherlands August 1, 2020; Norway August 1, 2020; Poland July 1, 2020; Portugal August 1, 2020; Romania June 1, 2020; Russia September 1, 2020; Serbia October 1, 2020; Slovakia August 1, 2020; Slovenia August 1, 2020; Spain July 1, 2020; Sweden September 1, 2020; Switzerland August 1, 2020; Ukraine June 1, 2020; United Kingdom August 1, 2020; Turkey August 1, 2020; Egypt October 1, 2020; Libya June 1, 2020; Morocco July 1, 2020; Syria June 1, 2020

Table S12: 28-day mortality in the second pandemic wave (versus the first wave) among total and hospitalized dialysis patients when considering end of the first wave on July 15, 2020 and the start of the second wave on August 15, 2020 (presented are hazard ratios with 95% confidence intervals)

**Total**	**Pandemic waves (N=2,966)**		
	**First wave (N=1,235)**	**Second wave (N=1,731)**	**p-value**
**Mortality, n (%)**	**299 (24.2)**	**342 (19.8)**	**0.004**
Model 1	Ref.	0.77 (0.66, 0.90)	0.001
Model 2	Ref.	0.69 (0.59, 0.81)	<0.001
Model 3	Ref.	0.74 (0.64, 0.87)	<0.001
Model 4	Ref.	0.77 (0.66, 0.91)	0.001
Model 5	Ref.	0.78 (0.67, 0.92)	0.004
Model 6	Ref.	0.93 (0.79, 1.10)	0.79
**Hospitalized**	**Pandemic waves (N=1,641)**		
	**First wave (N=877)**	**Second wave (N=764)**	**p-value**
**Mortality, n (%)**	**269 (30.7)**	**235 (30.8)**	**0.97**
Model 1	Ref.	0.96 (0.80, 1.14)	0.62
Model 2	Ref.	0.87 (0.73, 1.04)	0.12
Model 3	Ref.	0.90 (0.75, 1.07)	0.22
Model 4	Ref.	0.89 (0.75, 1.07)	0.22
Model 5	Ref.	0.89 (0.74, 1.07)	0.20

Model 1: crude

Model 2: Model 1 + age, and sex

Model 3: Model 2 + reason for screening, and presence of no to limited symptoms

Model 4: Model 3 + smoking, hypertension, diabetes mellitus, chronic lung disease, heart failure, chronic artery disease, auto-immune disease, malignancy, and frailty score

Model 5: Model 4 + cough, shortness of breath, fever, sore throat, O2 saturation, pulse, temperature, systolic blood pressure, diastolic blood pressure, lymphocytes, and c-reactive protein

Model 6: Model 5 + hospitalization

Table S13: 28-day mortality in the second pandemic wave (versus the first wave) among total and hospitalized kidney transplant recipients when considering end of the first wave on July 15, 2020 and the start of the second wave on August 15, 2020 (presented are hazard ratios with 95% confidence intervals)

**Total**	**Pandemic waves (N=1,011)**		
	**First wave (N=464)**	**Second wave (N=547)**	**p-value**
**Mortality, n (%)**	**89 (19.2)**	**72 (13.2)**	**0.009**
Model 1	Ref.	0.66 (0.48, 0.90)	0.008
Model 2	Ref.	0.80 (0.59, 1.10)	0.17
Model 3	Ref.	0.87 (0.63, 1.19)	0.38
Model 4	Ref.	0.83 (0.60, 1.14)	0.25
Model 5	Ref.	0.84 (0.60, 1.18)	0.31
Model 6	Ref.	0.98 (0.70, 1.37)	0.90
**Hospitalized**	**Pandemic waves (N=722)**		
	**First wave (N=390)**	**Second wave (N=332)**	**p-value**
**Mortality, n (%)**	**87 (22.3)**	**70 (21.1)**	**0.69**
Model 1	Ref.	0.92 (0.67, 1.27)	0.62
Model 2	Ref.	1.00 (0.73, 1.37)	0.98
Model 3	Ref.	1.03 (0.75, 1.42)	0.84
Model 4	Ref.	1.01 (0.73, 1.40)	0.96
Model 5	Ref.	1.00 (0.71, 1.42)	0.99

Model 1: crude

Model 2: Model 1 + age, and sex

Model 3: Model 2 + reason for screening, and presence of no to limited symptoms

Model 4: Model 3 + smoking, hypertension, diabetes mellitus, chronic lung disease, heart failure, chronic artery disease, auto-immune disease, malignancy, and frailty score

Model 5: Model 4 + cough, shortness of breath, fever, sore throat, O2 saturation, pulse, temperature, systolic blood pressure, diastolic blood pressure, lymphocytes, and c-reactive protein

Model 6: Model 5 + hospitalization

Table S14: 28-day mortality in the second pandemic wave (versus the first wave) in hospitalized and non-hospitalized dialysis patients by type of dialysis modality (presented are hazard ratios with 95% confidence intervals)

Total						
	Hemodialysis (N=2,712)			Peritoneal dialysis (N=196)		
	First wave (N=1,177)	Second wave (N=1,535)	p-value	First wave (N=69)	Second wave (N=127)	p-value
**Mortality, n(%)**	282 (24.0)	298 (19.4)	0.004	20 (29.0)	33 (26.0)	0.65
Model 1	Ref.	0.77 (0.65, 0.90)	<0.001	Ref.	0.83 (0.48, 1.45)	0.52
Model 2	Ref.	0.69 (0.59, 0.81)	<0.001	Ref.	0.71 (0.41, 1.25)	0.24
Model 3	Ref.	0.75 (0.63, 0.88)	<0.001	Ref.	0.71 (0.40, 1.26)	0.25
Model 4	Ref.	0.75 (0.64, 0.89)	<0.001	Ref.	NR	-
Model 5	Ref.	0.78 (0.66, 0.91)	0.004	Ref.	NR	-
Model 6	Ref.	0.91 (0.77, 1.08)	0.29	Ref.	NR	-
**In hospitalized patients**						
	Hemodialysis (N=1,541)			Peritoneal dialysis (N=124)		
	First wave (N=838)	Second wave (N=703)	p-value	First wave (N=52)	Second wave (N=72)	p-value
**Mortality, n(%)**	255 (30.4)	211 (30.0)	0.86	17 (32.7)	25 (34.7)	0.81
Model 1	Ref.	0.94 (0.78, 1.13)	0.52	Ref.	0.99 (0.53, 1.83)	0.97
Model 2	Ref.	0.86 (0.71, 1.03)	0.10	Ref.	0.92 (0.49, 1.72)	0.80
Model 3	Ref.	0.88 (0.74, 1.06)	0.18	Ref.	0.90 (0.47, 1.67)	0.76
Model 4	Ref.	0.86 (0.72, 1.04)	0.12	Ref.	NR	
Model 5	Ref.	0.86 (0.72, 1.04)	0.12	Ref.	NR	

NR: Not reliable, numbers of deaths too few to allow a valid multivariable adjustment.

Model 1: crude
Model 2: Model 1 + age, and sex
Model 3: Model 2 + reason for screening, and presence of no to limited symptoms
Model 4: Model 3 + smoking, hypertension, diabetes mellitus, chronic lung disease, heart failure, chronic artery disease, auto-immune disease, malignancy, and frailty score
Model 5: Model 4 + cough, shortness of breath, fever, sore throat, O2 saturation, pulse, temperature, systolic blood pressure, diastolic blood pressure, lymphocytes, and c-reactive protein
Model 6: Model 5 + hospitalization

Table S15: Baseline patient and disease characteristics by COVID-19 pandemic waves in hospitalized dialysis patients (N=1,668). (First wave: From March 1, 2020 to July 31, 2020. Second wave: from August 1, 2020 to February 28, 2021).

	**Pandemic waves**			
	**First wave (N=893)**	**Second wave (N=775)**	**p-value**	**Std. difference**
**Patient characteristics**				
Female, n (%)	337 (37.7)	272 (35.1)	0.26	0.05
Age (years)	67.0 (13.9)	69.3 (13.2)	0.001	-0.17
Body Mass Index, kg/m^2	26.5 (5.4)	26.8 (5.5)	0.32	-0.05
Caucasians, n (%)	768 (87.1)	629 (82.2)	0.01	0.13
Tobacco use, n (%)			0.13	0.12
Current	72 (8.1)	58 (7.5)		
Prior	192 (21.5)	203 (26.2)		
Never	392 (43.9)	332 (42.8)		
Unknown	237 (26.5)	182 (23.5)		
Reason for screening*, n(%)			0.04	0.15
Symptoms only, %	513 (67.5)	536 (69.2)		
Symptoms & COVID+ Contact,%	139 (18.3)	111 (14.3)		
COVID+ Contact only, %	62 (8.2)	58 (7.5)		
Routine, %	46 (6.1)	70 (9.0)		
Clinical frailty scale, AU	4.0 (1.8)	4.2 (1.8)	0.12	-0.09
Comorbidities, n (%)				
Hypertension	748 (83.8)	646 (83.4)	0.82	0.01
Diabetes Mellitus	386 (43.2)	387 (49.9)	0.01	-0.13
Coronary artery disease	305 (34.2)	321 (41.4)	0.002	-0.15
Heart failure	230 (25.8)	224 (28.9)	0.15	-0.07
Chronic lung disease	137 (15.3)	118 (15.2)	0.95	0.00
Active malignancy	65 (7.3)	50 (6.5)	0.51	0.03
Auto-immune disease,	39 (4.4)	37 (4.8)	0.69	-0.02
Primary kidney disease, n(%)				
Primary Glomerulonephritis	149 (16.9)	66 (8.6)	<0.001	0.25
Pyelonephritis	16 (1.8)	9 (1.2)	0.29	0.05
Interstitial nephritis	34 (3.9)	10 (1.3)	0.001	0.16
Hereditary kidney disease	55 (6.2)	30 (3.9)	0.03	0.10
Congenital diseases	10 (1.1)	9 (1.2)	0.93	0.00
Vascular diseases	118 (13.4)	147 (19.2)	0.001	-0.16
Sec. glomerular disease	64 (7.2)	24 (3.1)	<0.001	0.19
Diabetic kidney disease	221 (25.0)	233 (30.5)	0.01	-0.12
Other	141 (16.0)	165 (21.6)	0.003	-0.14
Unknown	75 (8.5)	71 (9.3)	0.57	-0.03
**Disease characteristics**				
Presenting symptoms, n (%)				
Sore throat	99 (11.1)	114 (14.7)	0.05	0.02
Cough	472 (55.5)	394 (51.6)	0.12	0.08
Shortness of breath	368 (43.1)	301 (39.3)	0.12	0.08
Fever	579 (67.1)	433 (57.0)	<0.001	0.21
Headache	91 (10.2)	75 (9.7)	0.78	-0.02
Nausea or vomiting	99 (11.1)	108 (14.0)	0.09	-0.01
Diarrhoea	112 (12.6)	120 (15.5)	0.13	0.04
Myalgia or arthralgia	184 (20.7)	200 (25.9)	0.01	-0.08
Limited to no symptoms, n (%)	93 (10.8)	116 (15.1)	0.01	0.13
Duration of dialysis (years)	5 (3, 8)	4 (4, 7)	<0.001	0.18
Vital signs				
Temperature, °C	37.6 (1.0)	37.5 (1.0)	0.19	0.07
Respiration rate, /min	20.0 (5.0)	19.0 (5.0)	<0.001	0.21
O2 saturation room air, %	93.0 (5.2)	92.9 (6.5)	0.74	0.02
Systolic BP, mm Hg	135.8 (25.8)	133.8 (25.5)	0.16	0.08
Diastolic BP, mm Hg	75.1 (15.4)	72.5 (15.6)	0.002	0.17
Pulse rate, BPM	84.0 (15.1)	82.6 (16.3)	0.12	0.09
Laboratory test results				
Lymphocytes, x1000/µL	0.9 (0.6, 1.4)	0.8 (0.6, 1.2)	0.03	0.01
CRP, mg/L	29 (7, 86)	40 (12, 99)	<0.001	-0.11

Continuous variables are reported as mean (standard deviation) or median (Inter quartile interval). Groups were compared using independent sample t-test, Mann-Whitney U-test, or Pearson Chi-square test as appropriate. Obesity is defined as BMI >30 kg/m^2. *Abbreviations are:* Std., standardized; BMI, body mass index; °C, degree Celsius; O2, oxygen; BP, blood pressure; BPM, beats per minute; eGFR, estimated glomerular filtration rate; CRP, C-reactive protein;

*Total number may not add up due to missing data

Table S16: Baseline patient and disease characteristics by COVID-19 pandemic waves in hospitalized kidney transplant recipients (First wave: From March 1, 2020 to July 31, 2020. Second wave: from August 1, 2020 to February 28, 2021)

	Pandemic waves (N=741)			
	First wave (N=399)	Second wave (N=342)	p-value	Std. difference
**Patient characteristics**				
Female, n (%)	156 (39.1)	132 (38.6)	0.89	0.01
Age (years)	59.2 (13.4)	59.0 (12.1)	0.84	0.02
Body Mass Index, kg/m^2	27.2 (4.9)	27.5 (5.3)	0.48	-0.06
Caucasians, n (%)	329 (87.0)	275 (81.4)	0.04	-0.16
Tobacco use, n (%)			0.29	0.14
Current	14 (3.5)	19 (5.6)		
Prior	94 (23.6)	81 (23.7)		
Never	205 (51.4)	157 (45.9)		
Unknown	86 (21.6)	85 (24.9)		
Reason for screening*, n(%)			<0.001	0.37
Symptoms only, %	272 (92.5)	277 (81.2)		
Symptoms & COVID+ Contact,%	15 (5.1)	40 (11.7)		
COVID+ Contact only, %	1 (0.3)	14 (4.1)		
Routine, %	6 (2.0)	10 (2.9)		
Clinical frailty scale, AU	3.0 (1.6)	3.2 (1.4)	0.10	-0.09
Comorbidities, n (%)				
Hypertension	333 (83.5)	287 (83.9)	0.87	-0.01
Diabetes Mellitus	126 (31.6)	124 (36.3)	0.18	-0.10
Coronary artery disease	85 (21.3)	69 (20.2)	0.71	0.03
Heart failure	38 (9.5)	45 (13.2)	0.12	-0.11
Chronic lung disease	34 (8.5)	33 (9.6)	0.59	-0.04
Active malignancy	25 (6.3)	11 (3.2)	0.05	0.14
Auto-immune disease,	23 (5.8)	17 (5.0)	0.63	0.04
Primary kidney disease, n(%)				
Primary Glomerulonephritis	65 (16.7)	56 (16.5)	0.94	0.00
Pyelonephritis	15 (3.9)	8 (2.4)	0.25	0.09
Interstitial nephritis	19 (4.9)	11 (3.2)	0.27	0.08
Hereditary kidney disease	55 (14.1)	44 (13.0)	0.65	0.03
Congenital diseases	13 (3.3)	13 (3.8)	0.72	-0.03
Vascular diseases	32 (8.2)	28 (8.3)	0.88	0.00
Sec. glomerular disease	17 (4.4)	22 (6.5)	0.20	-0.09
Diabetic kidney disease	53 (13.6)	32 (9.4)	0.08	0.13
Other	59 (15.2)	76 (22.4)	0.01	-0.19
Unknown	61 (15.7)	49 (14.5)	0.64	0.03
Immunosuppressants			0.03	0.21
Monotherapy	16 (4.1)	3 (0.9)		
Dual therapy	126 (31.9)	116 (34.3)		
Triple therapy	253 (64.1)	219 (64.8)		
**Disease characteristics**				
Presenting symptoms, n (%)				
Sore throat	55 (13.9)	65 (19.0)	0.03	
Cough	255 (64.7)	196 (59.4)	0.14	0.11
Shortness of breath	192 (49.0)	170 (51.1)	0.58	0.04
Fever	293 (74.6)	238 (71.0)	0.29	0.08
Headache	61 (15.4)	75 (22.1)	0.01	0.21
Nausea or vomiting	67 (16.9)	66 (19.4)	0.23	0.09
Diarrhoea	111 (28.0)	102 (29.8)	0.36	0.07
Myalgia or arthralgia	104 (26.2)	98 (28.7)	0.37	0.07
Limited to no symptoms, n (%)	6 (1.5)	14 (4.1)	0.03	0.16
Duration of tx. (years)			0.002	0.27
<1 year	26 (6.6)	49 (14.4)		
1-5 years	146 (36.9)	118 (34.7)		
>5 years	224 (56.6)	173 (50.9)		
Vital signs				
Temperature, °C	37.5 (1.1)	37.6 (1.1)	0.29	-0.08
Respiration rate, /min	21.4 (7.3)	21.2 (7.2)	0.77	0.02
O2 saturation room air, %	93.3 (8.2)	93.1 (8.5)	0.73	0.03
Systolic BP, mm Hg	131.3 (21.0)	134.0 (22.8)	0.15	-0.12
Diastolic BP, mm Hg	76.5 (13.8)	79.7 (15.1)	0.01	-0.22

Pulse rate, BPM	86.6 (16.9)	90.4 (17.2)	0.01	-0.22
Laboratory test results				
eGFR	39.2 (22.7)	41.6 (39.2)	0.67	-0.07
Lymphocytes, x1000/µL	0.8 (0.5, 1.2)	0.7 (0.5, 1.2)	0.26	-0.03
CRP, mg/L	53 (15, 104)	49 (13, 100)	0.35	0.08

Continuous variables are reported as mean (standard deviation) or median (Inter quartile interval). Groups were compared using independent sample t-test, Mann-Whitney U-test, or Pearson Chi-square test as appropriate. Obesity is defined as BMI >30 kg/m^2. *Abbreviations are:* Std., standardized; BMI, body mass index; °C, degree Celsius; O2, oxygen; BP, blood pressure; BPM, beats per minute; eGFR, estimated glomerular filtration rate; CRP, C-reactive protein;

*Total number may not add up due to missing data

Figure S1: Daily reported COVID-19 cases of dialysis patients (panel A, n=3,004) and kidney transplant recipients (panel B, n=1,035) between March 1st, 2020 and February 28th, 2021

A) Dialysis patients

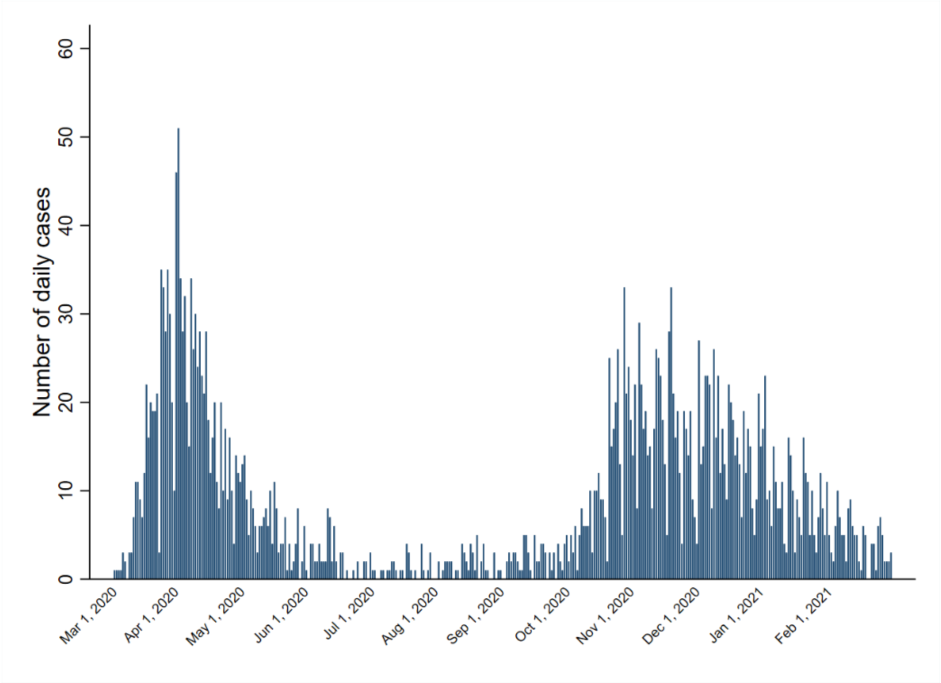

B) Kidney transplant recipients

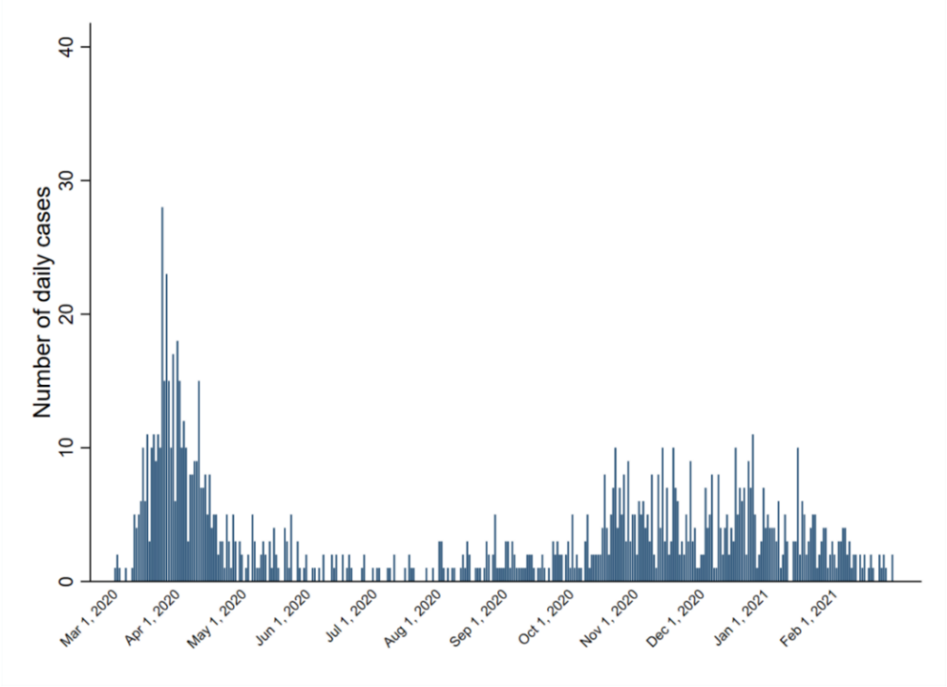

Figure S2: Kaplan-Meier curves for 28-day mortality by COVID-19 pandemic waves in the total population (left panels) and the population of hospitalized patients (right panels) for dialysis patients (upper panels) and kidney transplant recipients (lower panels)

A) Total dialysis patients

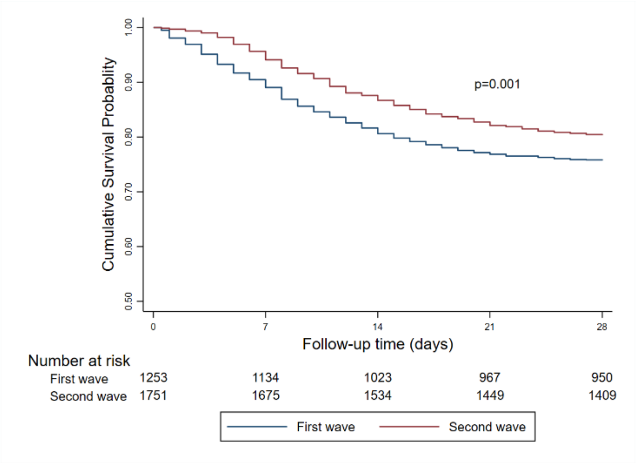

B) Hospitalized dialysis patients

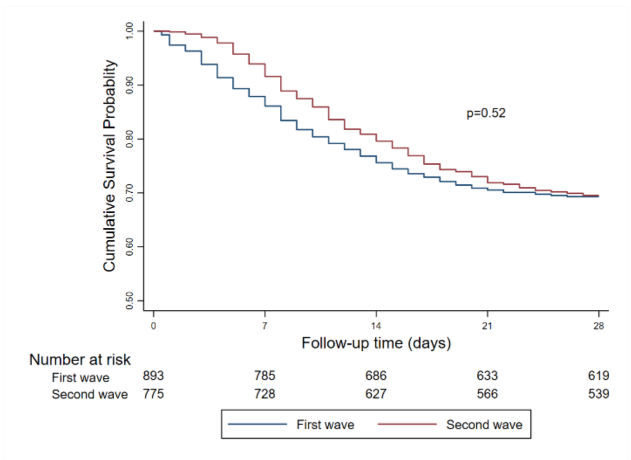

C) Total kidney transplant recipients

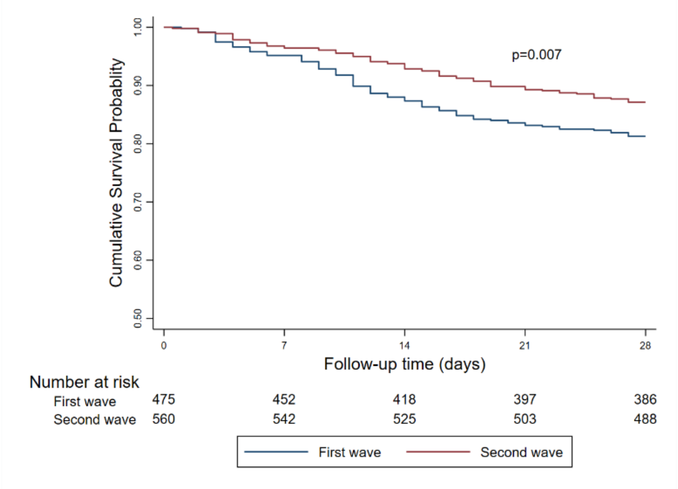

D) Hospitalized kidney transplant recipients

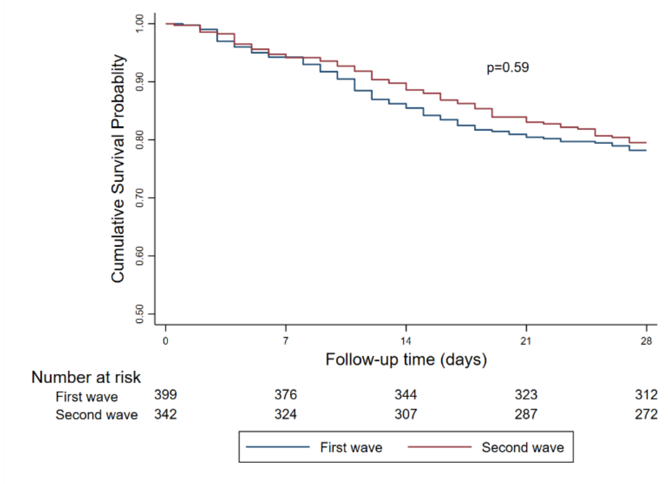

Figure S3: Kaplan-Meier curves for 3-month mortality by COVID-19 pandemic waves in the total population (left panels) and the population of hospitalized patients (right panels) for dialysis patients (upper panels) and kidney transplant recipients (lower panels)

A) Total dialysis patients

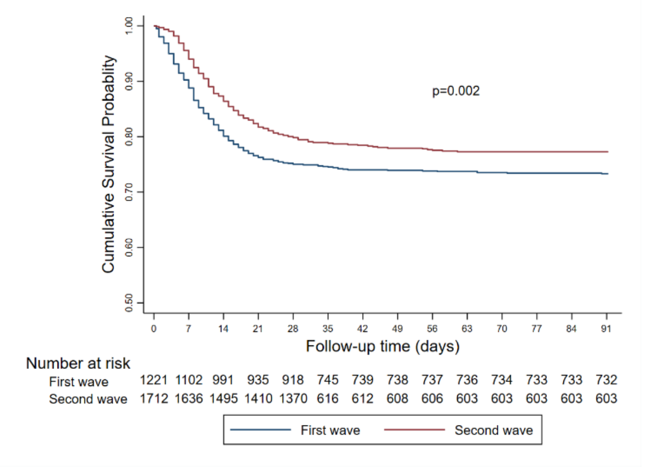

B) Hospitalized dialysis patients

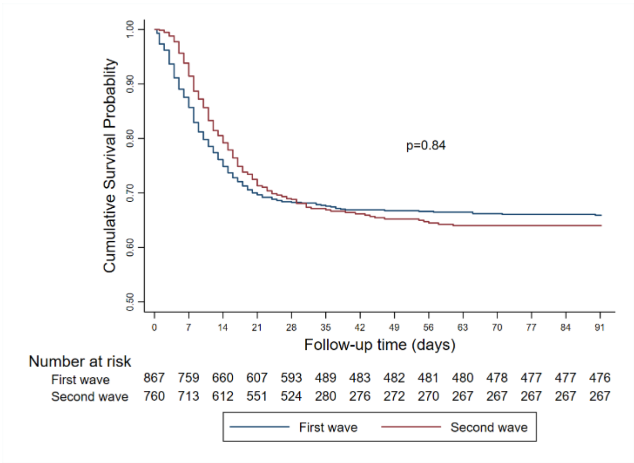

C) Total kidney transplant recipients

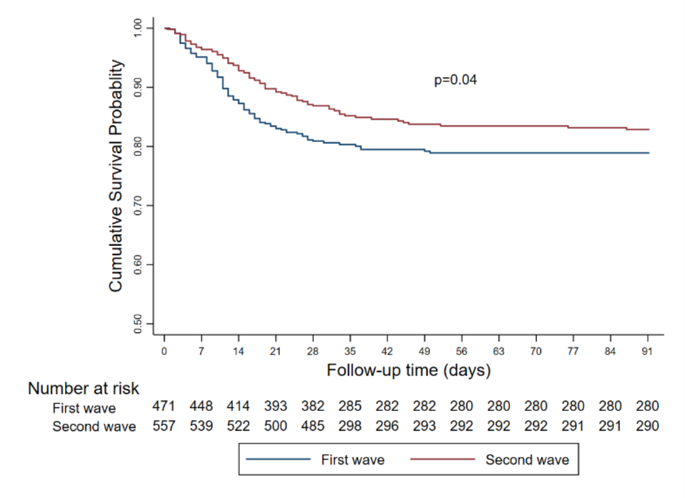

D) Hospitalized kidney transplant recipients

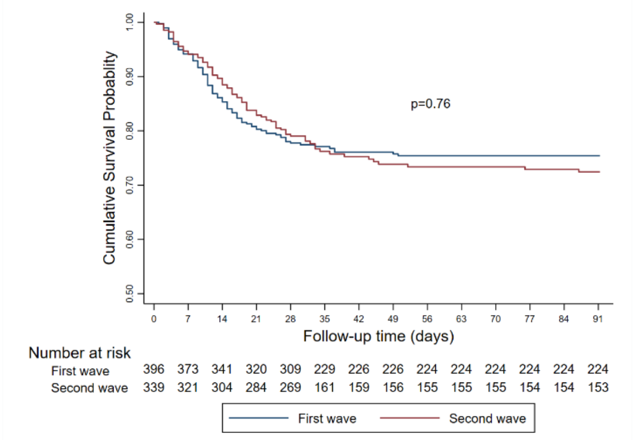

Figure S4: Kaplan-Meier curves for 28-day mortality by COVID-19 pandemic waves in the ICU admitted dialysis patients (left panel) and ICU admitted kidney transplant recipients (right panel)

A) From date of presentation in ICU dialysis patients

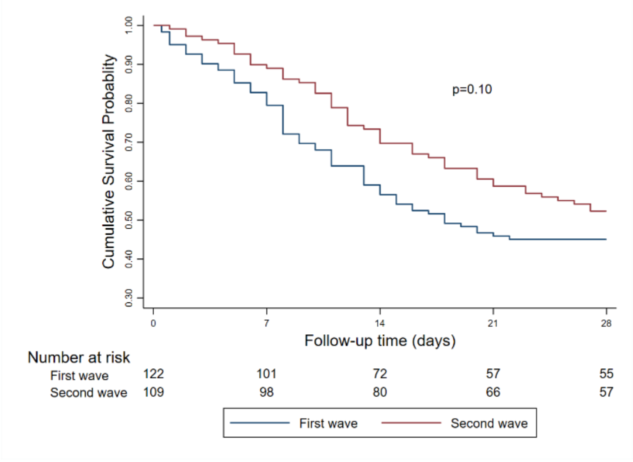

B) From date of symptoms onset in ICU dialysis patients

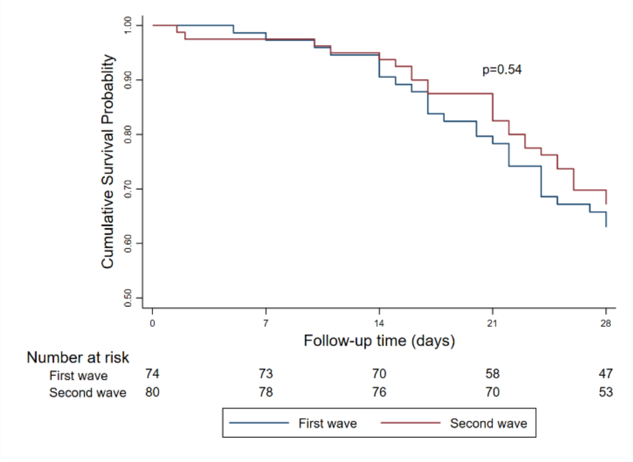

Figure S5: Association between pandemic waves (second vs. first) and 28-day mortality in dialysis patients across key subgroups (Presented hazard ratios are from fully adjusted model*)

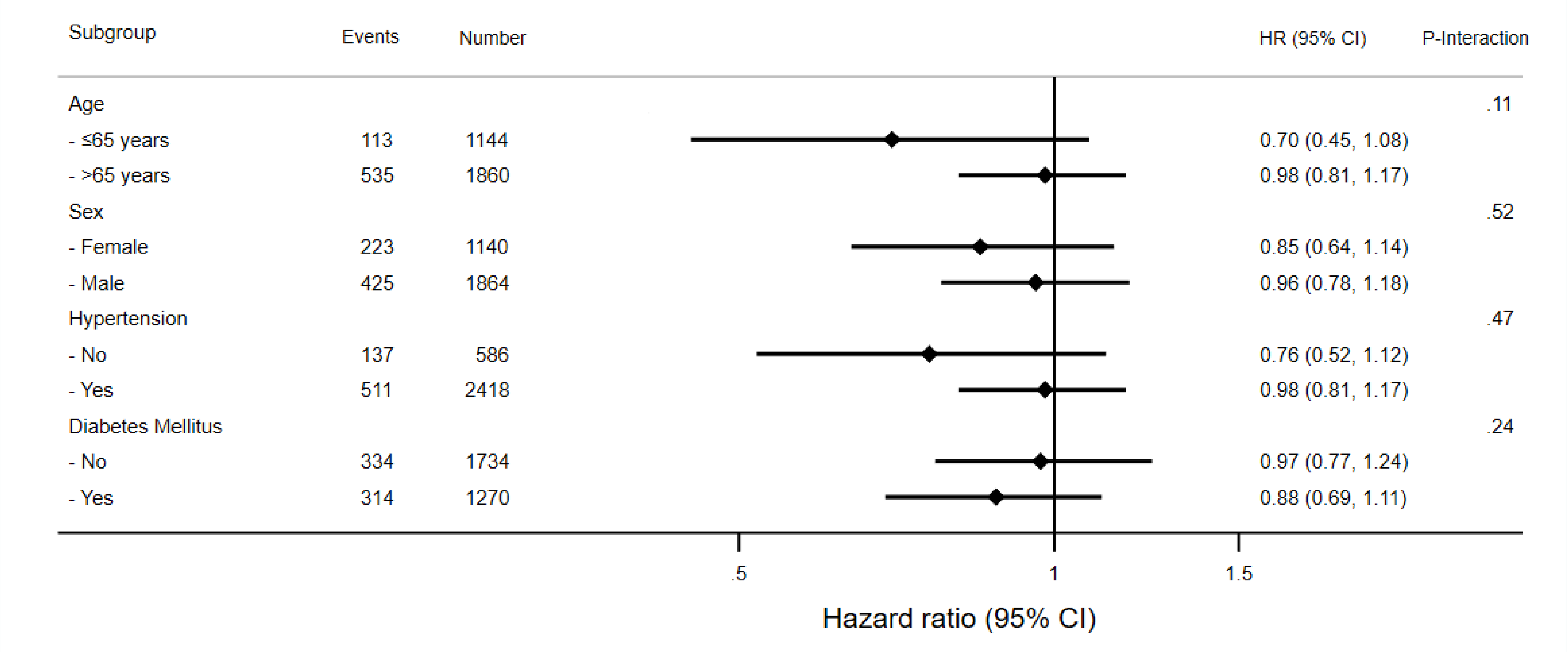

*****adjusted for age, sex, reason for screening, and presence of no to limited symptoms, smoking, hypertension, diabetes mellitus, chronic lung disease, heart failure, chronic artery disease, auto-immune disease, malignancy, and frailty score, cough, shortness of breath, fever, sore throat, O2 saturation, pulse, temperature, systolic blood pressure, diastolic blood pressure, lymphocytes, and c-reactive protein, hospitalization
(when analysing a subgroup, model was not adjusted for corresponding variable)

Figure S6: Association between pandemic waves (second vs. first) and 28-day mortality in kidney transplant recipients across key subgroups

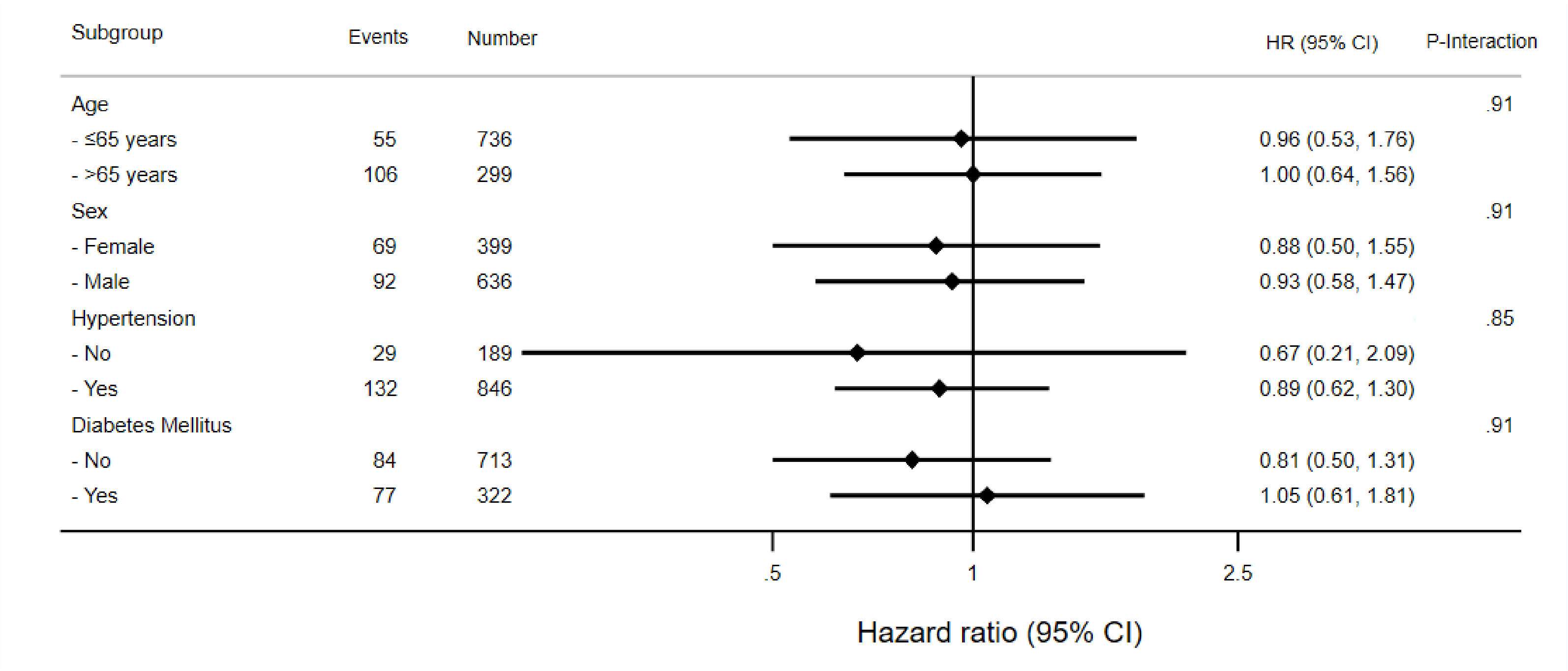

***adjusted for age, sex, reason for screening, and presence of no to limited symptoms, smoking, hypertension, diabetes mellitus, chronic lung disease, heart failure, chronic artery disease, auto-immune disease, malignancy, and frailty score, cough, shortness of breath, fever, sore throat, O2 saturation, pulse, temperature, systolic blood pressure, diastolic blood pressure, lymphocytes, and c-reactive protein, hospitalization

(when analysing a subgroup, model was not adjusted for corresponding variable)

Additional references

S1: Subramanian R, He Q, Pascual M. Quantifying asymptomatic infection and transmission of COVID-19 in New York City using observed cases, serology, and testing capacity. Proc Natl Acad Sci U S A. 2021;118(9):e2019716118.

S2: Ikizler TA, Kliger AS Minimizing the risk of COVID-19 among patients on dialysis. *Nat Rev Nephrol* **16,** 311–313 (2020).

S3: Williamson E, Walker AJ, Bhaskaran K. et al. Factors associated with COVID-19-related death using OpenSAFELY. *Nature* **584,** 430–436 (2020).

S4: https://www.nature.com/articles/d41586-020-02483-2. Accessed September 15, 2021

S5: Swedish Civil Contingencies Agency (MSB). National ban on visiting elderly homes. (2020).

S6: Public Health England. Guidance on shielding and protecting people who are clinically extremely vulnerable from COVID-19. (2020).

S7: World Health Organization. A coordinated global research roadmap: 2019 novel coronavirus. March 2020 (https://www.who.int/publications/m/item/a-coordinated-global-research-roadmap. opens in new tab).

S8: World Health Organization. R&D blueprint and Covid-19 (https://www.who.int/teams/blueprint/covid-19. opens in new tab).

S9: WHO Solidarity Trial Consortium, Pan H, Peto R, et al. Repurposed Antiviral Drugs for Covid-19 - Interim WHO Solidarity Trial Results. N Engl J Med. 2021;384(6):497-511.

S10: Kragholm K, Gerds TA, Fosbøl E, et al. Association Between Prescribed Ibuprofen and Severe COVID-19 Infection: A Nationwide Register-Based Cohort Study. Clin Transl Sci. 2020;13(6):1103-1107.

S11: Drake TM, Fairfield CJ, Pius R, et al. Non-steroidal anti-inflammatory drug use and outcomes of COVID-19 in the ISARIC Clinical Characterisation Protocol UK cohort: a matched, prospective cohort study. Lancet Rheumatol. 2021;3(7):e498-e506.

S12: Karruli A, Spiezia S, Boccia F, et al. Effect of immunosuppression maintenance in solid organ transplant recipients with COVID-19: Systematic review and meta-analysis. Transpl Infect Dis. 2021:e13595.

STROBE Statement—checklist of items that should be included in reports of observational studies

	Item No	Recommendation	Page No
**Title and abstract**	1	(*a*) Indicate the study's design with a commonly used term in the title or the abstract	-
		(*b*) Provide in the abstract an informative and balanced summary of what was done and what was found	NA
**Introduction**			
Background/rationale	2	Explain the scientific background and rationale for the investigation being reported	2
Objectives	3	State specific objectives, including any prespecified hypotheses	2
**Methods**			
Study design	4	Present key elements of study design early in the paper	Supplementary material
Setting	5	Describe the setting, locations, and relevant dates, including periods of recruitment, exposure, follow-up, and data collection	Supplementary material
Participants	6	(*a*) *Cohort study*—Give the eligibility criteria, and the sources and methods of selection of participants. Describe methods of follow-up<br>*Case-control study*—Give the eligibility criteria, and the sources and methods of case ascertainment and control selection. Give the rationale for the choice of cases and controls<br>*Cross-sectional study*—Give the eligibility criteria, and the sources and methods of selection of participants	Supplementary material
		(*b*) *Cohort study*—For matched studies, give matching criteria and number of exposed and unexposed<br>*Case-control study*—For matched studies, give matching criteria and the number of controls per case	Supplementary material
Variables	7	Clearly define all outcomes, exposures, predictors, potential confounders, and effect modifiers. Give diagnostic criteria, if applicable	Supplementary material
Data sources/ measurement	8*	For each variable of interest, give sources of data and details of methods of assessment (measurement). Describe comparability of	Supplementary material

		assessment methods if there is more than one group	
Bias	9	Describe any efforts to address potential sources of bias	Supplementary material
Study size	10	Explain how the study size was arrived at	NA
Quantitative variables	11	Explain how quantitative variables were handled in the analyses. If applicable, describe which groupings were chosen and why	Supplementary material
Statistical methods	12	(*a*) Describe all statistical methods, including those used to control for confounding	Supplementary material
		(*b*) Describe any methods used to examine subgroups and interactions	Supplementary material
		(*c*) Explain how missing data were addressed	Supplementary material
		(*d*) *Cohort study*—If applicable, explain how loss to follow-up was addressed<br>*Case-control study*—If applicable, explain how matching of cases and controls was addressed<br>*Cross-sectional study*—If applicable, describe analytical methods taking account of sampling strategy	Supplementary material
		(*e*) Describe any sensitivity analyses	

Continued on next page

Results

Participants	13*	(a) Report numbers of individuals at each stage of study—eg numbers potentially eligible, examined for eligibility, confirmed eligible, included in the study, completing follow-up, and analysed	Supplementary material
		(b) Give reasons for non-participation at each stage	
		(c) Consider use of a flow diagram	
Descriptive data	14*	(a) Give characteristics of study participants (eg demographic, clinical, social) and information on exposures and potential confounders	3-4
		(b) Indicate number of participants with missing data for each variable of interest	
		(c) *Cohort study*—Summarise follow-up time (eg, average and total amount)	
Outcome data	15*	*Cohort study*—Report numbers of outcome events or summary measures over time	3-4
		Case-control study—Report numbers in each exposure category, or summary measures of exposure	
		Cross-sectional study—Report numbers of outcome events or summary measures	
Main results	16	(*a*) Give unadjusted estimates and, if applicable, confounder-adjusted estimates and their precision (eg, 95% confidence interval). Make clear which confounders were adjusted for and why they were included	3-4
		(*b*) Report category boundaries when continuous variables were categorized	3-4
		(*c*) If relevant, consider translating estimates of relative risk into absolute risk for a meaningful time period	3-4
Other analyses	17	Report other analyses done—eg analyses of subgroups and interactions, and sensitivity analyses	3-4

Discussion

Key results	18	Summarise key results with reference to study objectives	4-5
Limitations	19	Discuss limitations of the study, taking into account sources of potential bias or imprecision. Discuss both direction and magnitude of any potential bias	4-5
Interpretation	20	Give a cautious overall interpretation of results considering objectives, limitations, multiplicity of analyses, results from similar studies, and other relevant evidence	4-5

Generalisability	21	Discuss the generalisability (external validity) of the study results	4-5
**Other information**			
Funding	22	Give the source of funding and the role of the funders for the present study and, if applicable, for the original study on which the present article is based	1

*Give information separately for cases and controls in case-control studies and, if applicable, for exposed and unexposed groups in cohort and cross-sectional studies.

Note: An Explanation and Elaboration article discusses each checklist item and gives methodological background and published examples of transparent reporting. The STROBE checklist is best used in conjunction with this article (freely available on the Web sites of PLoS Medicine at http://www.plosmedicine.org/, Annals of Internal Medicine at http://www.annals.org/, and Epidemiology at http://www.epidem.com/). Information on the STROBE Initiative is available at www.strobe-statement.org.
